# Do we really apply evidence-based-recommendations to spine surgery? Results of an international survey

**DOI:** 10.1007/s10143-024-02502-0

**Published:** 2024-06-10

**Authors:** Ismail Bozkurt, Matthew W. Holt, Eric C. Robinson, Bipin Chaurasia, Mehmet Zileli

**Affiliations:** 1 Department of Neurosurgery, Medical Park Ankara Hospital, Ankara, Turkey; 2 Department of Neurosurgery, School of Medicine, Yuksek Ihtisas University, Ankara, Turkey; 3 Department of Natural Sciences, University of South Carolina Beaufort, Bluffton, SC USA; 4 Ross University School of Medicine, Miramar, FL 33027 USA; 5 Department of Neurosurgery, Neurosurgery Clinic, Birgunj, Nepal; 6 Department of Neurosurgery, Sanko University, Gaziantep, Turkey

**Keywords:** Spine, Surgery, Evidence-based medicine, Survey, Medical education

## Abstract

**Objective:**

This international survey investigated Evidence-Based Medicine (EBM) in spine surgery by measuring its acceptance among spine surgeons. It assessed their understanding of EBM and how they apply it in practice by analyzing responses to various clinical scenarios..

**Materials and methods:**

Following the CHERRIES guidelines, an e-survey was distributed to multiple social media forums for neurosurgeons and orthopedic surgeons on Facebook, LinkedIn, and Telegram and circulated further through email via the authors’ network. Three hundred participants from Africa, Asia, Europe, North America, and Oceania completed the survey.

**Results:**

Our study revealed that 67.7% (*n* = 203) of respondents used EBM in their practice, and 97.3% (*n* = 292) believed training in research methodology and EBM was necessary for the practice of spine surgery. Despite this endorsement of using EBM in spine surgery, we observed varied responses to how EBM is applied in practice based on example scenarios. The responders who had additional training tended to obey EBM guidelines more than those who had no additional training. Most surgeons responded as always or sometimes prescribing methylprednisolone to patients with acute spinal cord injury. Other significant differences were identified between geographical regions, training, practice settings, and other factors.

**Conclusions:**

Most respondents used EBM in practice and believed training in research methodology and EBM is necessary for spine surgery; however, there were significant variations on how to use them per case. Thus, the appropriate application of EBM in clinical settings for spinal surgery must be further studied.

**Supplementary Information:**

The online version contains supplementary material available at 10.1007/s10143-024-02502-0.

## Introduction

Evidence-based medicine (EBM) is the application of clinical methodology and decision-making thoroughly tested by duly controlled peer-reviewed studies. With the exponentially increasing number of publications, EBM aims to shift the traditional paradigm of making decisions based purely on the judgment of the physician or the experience of senior doctors to the use of results obtained from systematic and unbiased research in a patient-centered diagnosis and treatment model with the highest available evidence [1]. The definition of EBM has evolved, but the most widely accepted definition was from Sackett et al. in 1996 and revised in 2000. In summary, it stated that clinicians should use the best, unbiased, clinically sound, and up-to-date data available on a patient basis in prevention, diagnosis, and treatment [2, 3].

Neurosurgery and Orthopedic Surgery are the pioneers of spine surgery throughout the world. Neurosurgeons have utilized EBM and clinical guidelines to make sound decisions for the last three decades. However, the increasing number of guidelines have questioned the validity of these recommendations as they may stray away from the rigorous process outlined by The Institute of Medicine (IOM) [4]. Orthopedic Surgery also recognized the importance of EBM in the early 2000s, and journal sections reserved for “Evidence-Based Orthopedics” emerged [4]. The classical paradigm of trying non-operative treatment first and operative treatment, if the first step fails, has shifted towards surgical treatment vs. other treatment options.

Evidence-Based Spine Surgery (EBSS) has also emerged parallel to the development of EBM. The North American Spine Society (NASS) and Congress of Neurological Surgeons (CNS) frequently publish guidelines with evidence-based suggestions to provide the highest evidence-based decisions in spinal pathologies. For example, EBSS supported by randomized controlled trials has shown discectomy for symptomatic lumbar disc herniation (LDH), decompression of spinal stenosis, and decompression with fusion of degenerative spondylolisthesis offer clinical benefit in relieving lower back and radicular pain compared to conservative treatment [5]. However, pursuing EBSS as the sole deciding factor for diagnosing and treating spinal pathologies is unrealistic. Most of the publications on spine surgery are case-controlled retrospective cohorts or case series. Thus, the number of high-quality evidence parallel to spine surgery development is lacking [5]. Considering the value of EBM and the probability of further implementation into decision-making, this study sheds light on the reception and application of EBM by spinal surgeons and the use of evidence-based modalities in their clinical practice.

## Materials and methods

The cross-sectional and descriptive online survey consisted of three parts with 29 items. The survey was created using Google Forms (https://forms.gle/zLukb428wc8PtvoJ7). The most commonly used tests to evaluate EBM are Fresno, ACE, and Berlin, but they require a longer testing period, leaving a high frequency of blank questions. Thus, questions from previous EBM studies were reformatted into a spine surgery perspective to allow for a higher response rate. The CHERRIES guidelines for e-surveys were adhered to [6]. This guideline contains a detailed checklist to apply when conducting web-based surveys. The checklist serves to prevent a single user from filling in the same questionnaire multiple times, provide proper survey administration, and perform consistency or completeness checks before the questionnaire is submitted, etc. The link to the survey was deposited onto the largest social media group for neurosurgery, Neurosurgery Cocktail (NC). Platforms such as Facebook, LinkedIn, and Telegram were used. The survey was also posted on orthopedic/spine surgery social media groups and communities to ensure contact with orthopedic surgeons. The authors of this manuscript also circulated the open survey via email and posted the survey link to other neurosurgical/orthopedic/spine interest groups on various social media platforms. To increase the survey’s reach and interest, #neurosurgery, #orthopedics, #evidence-based medicine, and #spine hashtags were used. No ethical approval was obtained due to the anonymized nature of data collection.

### Data collection

The preliminary survey consisted of 35 items distributed to 5 participants. However, the length of the survey was a concern amongst participants, and keeping in mind the survey fatigue [7], it was reduced to 29 items in three parts. Thereafter, the survey was tested on a small group of neurosurgeons and orthopedic surgeons, whose suggestions were used to strengthen the survey and improve understandability. The preliminary responses were not included in the final data collection.

Part one of this survey consisted of eight questions mainly focused on demographic data. Part two evaluated the participant’s overall understanding of EBM with eight multiple-choice and three Likert scale questions [8, 9]. Part three consisted of 10 questions to assess the participant’s approach to specific clinical scenarios [10–14]. A brief description of the survey and the names of the investigators were provided at the start of the survey. No incentives were provided, and all participation was voluntary; reminders to complete the survey were sent out bi-monthly. Results were collected between November 1, 2022, and March 1, 2023. To complete the survey, participants logged in to their Google accounts. Thus, one response per participant was received.

### Statistical analysis

Data analysis was performed with SPSS 27.0, and a 95% confidence level was employed. Mean and standard deviation statistics are given for the measurements. The following tests were used to compare the significance levels between the groups: the independent-samples t-test for two groups, the one-way ANOVA test for more than two groups, and the Chi-square test for the relationship of grouped variables. All data not included in the results section, is supplied via the supplementary material.

## Results

### Demographics

Three hundred participants completed the survey, with a male predominance of 88%. Most respondents were in the 31–40 age group (46%), followed by the 41–50 age group (27.3%). Most participants were from Asia (50%), followed by Europe (20%) and North & South America (17.3%). A vast majority of the participants were neurosurgeons (95.7%), while most of them were consultants (48.3%). When evaluating the clinical practice setting, most participants stated they worked in a hospital (55.3%), while 60% said they did not receive additional training for spine surgery. The varying attributes of our survey participants are given in Table 1.

**Table 1:** Characteristics of the Responders to the Survey

Age		Gender	
< 30	39 (13.0%)	Male	264 (88.0%)
31–40	138 (46.0%)	Female	36 (12.0%)
41–50	82 (27.3%)	Specialty	
51–60	31 (10.3%)	Neurosurgeon	287 (95.7%)
61–70	9 (3.0%)	Orthopedics	7 (2.3%)
> 70	1 (0.3%)	Spine	0 (0.0%)
Region		Other	4 (1.3%)
North America	19 (6.3%)	Resident	84 (28.0%)
South America	33 (11.0%)	Setting	
Europe	60 (20.0%)	Academic practice	86 (28.7%)
Asia-Oceania	150 (50.0%)	Hospital employment	166 (55.3%)
Africa	37 (12.3%)	Private practice	41 (13.7%)
Oceania	1 (0.3%)	Military	2 (0.7%)
Years of medical practice		Other	5 (1.7%)
0–5 year	74 (24.7%)	Currently training	33 (11.0%)
6–10 year	56 (18.7%)	Status	
11–15 year	62 (20.7%)	Resident	84 (28.0%)
16–20 year	20 (10%)	Consultant	145 (48.3%)
> 20 year	45 (15.0%)	Academic	52 (17.3%)
Additional Training		Other	19 (6.3%)
Yes	120 (40.0%)		
No	180 (60.0%)		

### General evidence-based knowledge

We designed the second section of the survey to depict the participants’ understanding of EBM. Table 2 summarizes the most frequent responses to general EBM concepts. Most participants (67.7%) understand and use EBM in their practice, while a majority (71%) define EBM as combining personal experience with evidence derived from scientific research. The definition of randomization had a high rate of correct answers, with 66.3%, while the definition of “double-blind” in a randomized controlled study (RCT) received correct responses but at a lower rate of 33.3%. 86% of the responders have accepted that training for research methodology and evidence-based medicine is necessary for spine surgery, and an additional 11.3% partially believe. Meanwhile, 91% of the participants stated that they either always or frequently consider the level of evidence and grade of recommendation when analyzing the results of a published study. However, 82.0% of participants indicated that EBM is used to make correct and scientific medical decisions for the patient’s benefit.

**Table 2:** Most Frequent Responses in the Assessment of EBM

		*n*	%
Are you familiar with the concept “evidence-based medicine” (EBM)?	I know and understand EBM but do not use it	64	21.3
	I know and understand EBM and use it during my practice	203	67.7
Which of the following definitions do you think BEST defines “evidence-based medicine”?	Exclusive use of medical research results in medical decision.	60	20.0
	Combining personal clinical experience with the best evidence derived from medical scientific knowledge to reach a medical decision.	213	71.0
Which of the following statements is FALSE concerning “VALIDITY”?	Validity refers to how well the results represent true findings outside the study.	80	26.7
	Internal and external validity can be both performed independent of each other.	72	24.0
	I do not know	87	29.0
How would you define RANDOMIZATION?	Treatment and control patients are followed an equal amount of time and treated the same	73	24.3
	Both known and unknown prognostic factors are equally balanced between treatment and control patient groups	199	66.3
In a randomized controlled study, how would you define double-blind?	Patients and researchers are blind	100	33.3
	Patients, researchers and outcome assessors are blind	65	21.7
Do you believe a training for research methodology and evidence based medicine is necessary in spine surgery?	Yes	258	86.0
	Partially	34	11.3
Do you take into consideration the “level of evidence” or “grade of recommendation” provided by the study when you are reading a published scientific article?	Always	106	35.3
	Frequently	167	55.7
Evidence-based medicine is used to.	Make medical decisions correctly and scientifically for the patient’s benefit	246	82.0
	Know the correct form of a manuscript	20	6.7

### Various clinical scenarios

Table 3 summarizes the responses to the applicability of various medical approaches when making decisions about spinal pathologies. The rate of those who always or frequently refer to guidelines or scoring systems in their decisions was (94.3%). When no guideline or scoring system is used, the most commonly referred methods are “Literature search” and “Personal experience.”

In patients with severe back pain, the most preferred initial approach chosen by participants was “Obtain MRI”. In addition, there is a common belief that the spinal surgical approach and the addition of arthrodesis can improve clinical outcomes. There are some different opinions about whether to treat patients with low bone mineral density (BMD T <-2.5) before spine surgery, but there seems to be a trend to treat sometimes or always. There are also various opinions on prescribing high-dose methylprednisolone to patients with acute spinal cord injury. However, the rate of those who always or sometimes apply this treatment is relatively high. Most respondents stated that ACDF alone is sufficient in correcting sagittal alignment and that anterior discectomy has better outcomes than posterior when treating cervical radiculopathy. A majority of participants (68.3%) recommended physical and medical therapy to a patient with disc herniation in the absence of leg pain, whereas only 16% opted for no treatment.

**Table 3:** Most Frequent Answers to Various Clinical Scenarios

		*n*	%
Do you adhere to any guidelines or scoring systems when making decisions in spinal pathologies?	Always	132	44.0
	Frequently	151	50.3
	Rarely	12	4.0
	Never	5	1.7
In patients where no guideline or scoring system is used, what is your decision mostly based on? (Choose all that apply)	Personal experience	148	49.3
	Face-to-face consultation with colleagues	133	44.3
	Literature search	165	55.0
	Reference text-books	98	32.7
In a patient applying to your clinic with severe low back pain that has affected their quality of life with no red-flag signs detected during examination. What is your FIRST step?	Obtain X-ray/CT	69	23.0
	Obtain MRI	109	36.3
	Prescribe medication	93	31.0
Do you believe the choice of surgical approach improves clinical outcomes in patients with thoracic and lumbar fractures?	Always	116	38.7
	Sometimes	163	54.3
	Never	9	3.0
	Not Sure	12	4.0
Do you believe the addition of arthrodesis to instrumented fixation improves outcomes in patients with thoracic and lumbar burst fractures?	Always	91	30.3
	Sometimes	176	58.7
Do you treat low BMD (<-2.5) before spine surgery?	Always	83	27.7
	Sometimes	158	52.7
	Never	36	12
	Not Sure	23	7.7
Do you prescribe high dose methylprednisolone to patients presenting with acute spinal cord injury with neurological findings?	Always	100	33.6
	Sometimes	105	35.2
	Never	83	27.9
Do you perform fusion in patients treated for lumbar stenosis regardless of the presence of spondylolisthesis after decompression?	Always	54	18.0
	Sometimes	205	68.3
Which of the following statements about the surgical treatment of cervical radiculopathy do you agree with the most?	When correcting cervical sagittal alignment. ACDF alone is sufficient.	100	33.3
	Anterior surgery results in better outcomes than posterior surgery	89	29.7
What is your recommendation to a patient with unilateral extruded disc herniation with no leg pain at the time of your examination?	Microdiscectomy	47	15.7
	Physical therapy	93	31.0
	Medical therapy	112	37.3
	No treatment	48	16.0

### Group comparisons

#### Gender

Since only a few women (12%) completed the survey, our authors avoided making conclusions about gender-wise comparisons from this data. The rate of those who know and understand EBM and use it in practice is relatively high in both men (67.0%) and women (72.2%). In the question about what is the best definition of EBM, the majority of both men (71.6%) and women (66.7%) participants gave the answer “combining personal clinical experience with the best evidence derived from medical, scientific research.” In the case of decision-making in patients where no guideline or scoring system is used, the highest percentage relies on literature searches, with 54.5% for men and 58.3% for women. However, we observed a striking statistical difference concerning the opinions on the surgical treatment of cervical radiculopathy is the higher agreement of women with the statement “The Neck Disability Index, SF-36, SF-12, and VAS are recommended outcome measures” compared to men (41.7% versus 20.8%, *p* = 0.013).

#### Age

Four groups were defined when analyzing results according to age (≤ 30, 31–40, 41–50, and 50 > years). The majority of all age groups have stated that they understand EBM and use it in their practice with the highest percentage. Table 4 displays responses to a broad range of issues, from adherence to specific guidelines or scoring systems in making decisions in spinal pathologies to the suggested treatment in certain situations, according to participants from four different age groups. A general table review shows significant differences in some questions between age groups. These findings indicate that other age groups perceive these issues differently, and their approaches to specific patient groups differ. However, it is essential to note that these differences are generally insignificant since most of the *p*-values across age groups were above 0.05.

**Table 4:** Comparison of Age Groups in Various Clinical Scenarios

		Age (years)								X^2^	p
		≤ 30		31–40		41–50		50>			
		n	%	n	%	n	%	n	%		
Do you adhere to any guidelines or scoring systems when making decisions in spinal pathologies?	Always	20	52.6	67	48.9	33	40.7	12	30.8	7.026	0.318
	Frequently	17	44.7	63	46.0	46	56.8	25	64.1		
	Rarely	1	2.6	7	5.1	2	2.5	2	5.1		
In patients where no guideline or scoring system is used, what is your decision mostly based on?	Personal experience	24	61.5	56	40.6	47	57.3	21	51.2	8.704	**0.033**
	Face-to-face consultation with colleagues	26	66.7	61	44.2	30	36.6	16	39.0	10.346	**0.016**
	Social media collaboration with colleagues	6	15.4	18	13.0	12	14.6	5	12.2	0.283	0.963
	Literature search	21	53.8	81	58.7	42	51.2	21	51.2	1.493	0.684
	Reference text-books	13	33.3	50	36.2	24	29.3	11	26.8	1.871	0.600
In a patient applying to your clinic with severe low back pain that has affected their quality of life with no red-flag signs detected during examination, what is your FIRST step?	Obtain X-ray/CT	11	28.2	35	25.4	17	20.7	6	14.6	12.311	0.421
	Obtain MRI	14	35.9	43	31.2	33	40.2	19	46.3		
	Prescribe medication	10	25.6	49	35.5	24	29.3	10	24.4		
	Suggest bed-rest	1	2.6	8	5.8	3	3.7	4	9.8		
	None	3	7.7	3	2.2	5	6.1	2	4.9		
Do you believe the choice of surgical approach improves clinical outcomes in patients with thoracic and lumbar fractures?	Always	15	38.5	54	39.1	30	36.6	17	41.5	8.451	0.489
	Sometimes	22	56.4	70	50.7	49	59.8	22	53.7		
	Never	0	0.0	5	3.6	2	2.4	2	4.9		
	Not Sure	2	5.1	9	6.5	1	1.2	0	0.0		
Do you believe the addition of arthrodesis to instrumented fixation improves outcomes in patients with thoracic and lumbar burst fractures?	Always	5	12.8	41	29.7	30	36.6	15	36.6	19.208	**0.023**
	Sometimes	29	74.4	80	58.0	49	59.8	18	43.9		
	Never	2	5.1	6	4.3	2	2.4	1	2.4		
	Not Sure	3	7.7	11	8.0	1	1.2	7	17.1		
Do you treat low BMD (T<-2.5) before spine surgery?	Always	18	46.2	33	23.9	25	30.5	7	17.1	18.407	**0.031**
	Sometimes	12	30.8	80	58.0	39	47.6	27	65.9		
	Never	3	7.7	17	12.3	12	14.6	4	9.8		
	Not Sure	6	15.4	8	5.8	6	7.3	3	7.3		
Do you prescribe high dose methylprednisolone to patients presenting with acute spinal cord injury with neurological findings?	Always	12	30.8	52	38.2	23	28.0	13	31.7	10.418	0.318
	Sometimes	19	48.7	47	34.6	26	31.7	13	31.7		
	Never	6	15.4	33	24.3	30	36.6	14	34.1		
	Not Sure	2	5.1	4	2.9	3	3.7	1	2.4		
Do you perform fusion in patients treated for lumbar stenosis with or without spondylolisthesis after decompression?	Always	6	15.4	30	21.7	12	14.6	6	14.6	19.786	**0.019**
	Sometimes	25	64.1	93	67.4	57	69.5	30	73.2		
	Never	3	7.7	12	8.7	13	15.9	3	7.3		
	Not Sure	5	12.8	3	2.2	0	0.0	2	4.9		
Which of the following statements about the surgical treatment of cervical radiculopathy do you agree with the most?	When correcting cervical sagittal alignment. ACDF alone is sufficient.	11	28.2	45	32.6	28	34.1	16	39.0	6.408	0.698
	Anterior surgery results in better outcomes than posterior surgery	11	28.2	46	33.3	18	22.0	14	34.1		
	TDA results in better outcomes than ACDF when treating single level soft disc herniations.	6	15.4	17	12.3	14	17.1	4	9.8		
	The Neck Disability Index. SF-36. SF-12 and VAS are recommended outcome measures for assessing…	11	28.2	30	21.7	22	26.8	7	17.1		
What is your recommendation to a patient with unilateral extruded disc herniation with no leg pain at the time of your examination?	Microdiscectomy	6	15.4	20	14.5	12	14.6	9	22.0	4.896	0.843
	Physical therapy	14	35.9	41	29.7	27	32.9	11	26.8		
	Medical therapy	16	41.0	52	37.7	31	37.8	13	31.7		
	No treatment	3	7.7	25	18.1	12	14.6	8	19.5		

#### Job status

The participants were divided into four groups according to their status: residents, consultants, academic personnel, and others. When evaluating techniques to conceal randomization, two significant differences were observed: calling a separate center via telephone to obtain the next patient allocation (F = 5.213, *p* = 0.002) and using opaque envelopes that contain the next treatment allocation (F = 2.708, *p* = 0.045). Using a coin toss was the most preferred method for randomization amongst all groups. In all groups, the proportion of those who always or frequently adhere to a guideline or scoring system when making decisions in spinal pathologies is relatively high. When they do not use a guideline or scoring system, most participants base their decisions on personal experiences or face-to-face consultations. The data above are a general trend in all groups, with no significant difference (*p* = 0.220 and *p* = 0.059). In clinical scenarios, the only statistically significant result was whether fusion is performed in patients with lumbar stenosis after decompression; all groups responded predominantly “always” or “sometimes” (*p* = 0.045).

#### Region of practice

Five different geographical regions (North America, South America, Europe, Asia, and Africa) were considered. Oceania respondent data was not compared for this region analysis since only one participant responded. Most participants from all regions reported knowing and applying EBM in their practice. However, the understanding and implementation of EBM rates are highest in North America and lower in Africa (X2 = 23.100, *p* = 0.027). These differences may point to insufficiencies in the education and application of EBM in certain regions. However, the majority opinion is that the best definition of EBM combines personal clinical experience with the best evidence derived from medical scientific research. The share of this view is higher in North America and Europe than in Asia and Africa. While assessing the opinion on the validity of the study designs, Asian and African continents had a higher trust rate in case reports than respondents from the American and European continents (*p* = 0.041).

Based on Table 5, there are regional differences regarding the practices of spinal pathologies. For instance, decision-making in spinal pathologies varies significantly based on the region of practice. A statistically significant difference was observed in face-to-face consultation with colleagues as a decision-making factor (*p* = 0.011) and literature search (*P* < 0.0001). Moreover, critical regional differences were observed in the initial management steps of severe low back pain patients (*p* = 0.005). The most common first step in North America and Europe is to obtain an MRI; in Asia and Africa, obtaining an X-ray/CT is more prevalent. Treating low BMD before spine surgery had the lowest rate, with African participants responding with Never and Not Sure at 32.4% (*p* = 0.001). Prescribing high-dose methylprednisolone to patients with acute spinal cord injury with neurological findings was a frequently practiced method in the European, Asian, and African continents (*p* = 0.002). Performing fusion in patients treated for lumbar stenosis was the lowest in the North American European continents (*p* = 0.006).

**Table 5:** Comparison of Region of Practice in Various Clinical Scenarios

		Region of practice										X^2^	p
		North America		South America		Europe		Asia		Africa			
		n	%	n	%	n	%	n	%	n	%		
Do you adhere to any guidelines or scoring systems when making decisions in spinal pathologies?	Always	8	44.4	9	27.3	23	39.0	74	50.0	18	50.0	12.004	0.151
	Frequently	10	55.6	23	69.7	31	52.5	68	45.9	18	50.0		
	Rarely	0	0.0	1	3.0	5	8.5	6	4.1	0	0.0		
In patients where no guideline or scoring system is used, what is your decision mostly based on?	Personal experience	12	63.2	11	33.3	30	50.0	73	48.7	22	59.5	6.384	0.172
	Face-to-face consultation with neurosurgeons	10	52.6	16	48.5	37	61.7	53	35.3	16	43.2	13.013	**0.011**
	Social media collaboration with neurosurgeons	1	5.3	1	3.0	6	10.0	26	17.3	7	18.9	7.538	0.110
	Literature search	14	73.7	18	54.5	47	78.3	68	45.3	18	48.6	22.16	**0.000**
	Reference text-books	8	42.1	6	18.2	20	33.3	52	34.7	12	32.4	4.194	0.380
In a patient applying to your clinic with severe low back pain that has affected their quality of life with no red-flag signs detected during examination, what is your FIRST step?	Obtain X-ray/CT	4	21.1	6	18.2	5	8.3	41	27.3	12	32.4	34.295	**0.005**
	Obtain MRI	8	42.1	10	30.3	30	50.0	53	35.3	8	21.6		
	Prescribe medication	5	26.3	13	39.4	21	35.0	39	26.0	15	40.5		
	Suggest bed-rest	0	0.0	0	0.0	1	1.7	13	8.7	2	5.4		
	None	2	10.5	4	12.1	3	5.0	4	2.7	0	0.0		
Do you believe the choice of surgical approach improves clinical outcomes in patients with thoracic and lumbar fractures?	Always	8	42.1	10	30.3	16	26.7	67	44.7	15	40.5	15.479	0.216
	Sometimes	11	57.9	21	63.6	41	68.3	72	48.0	18	48.6		
	Never	0	0.0	2	6.1	0	0.0	4	2.7	2	5.4		
	Not Sure	0	0.0	0	0.0	3	5.0	7	4.7	2	5.4		
Do you believe the addition of arthrodesis to instrumented fixation improves outcomes in patients with thoracic and lumbar burst fractures?	Always	8	42.1	10	30.3	8	13.3	53	35.3	11	29.7	20.456	**0.059**
	Sometimes	9	47.4	23	69.7	44	73.3	79	52.7	21	56.8		
	Never	1	5.3	0	0.0	1	1.7	6	4.0	3	8.1		
	Not Sure	1	5.3	0	0.0	7	11.7	12	8.0	2	5.4		
Do you treat low BMD (T <-2.5) before spine surgery?	Always	3	15.8	17	51.5	5	8.3	49	32.7	9	24.3	34.590	**0.001**
	Sometimes	14	73.7	9	27.3	40	66.7	78	52.0	16	43.2		
	Never	1	5.3	4	12.1	10	16.7	12	8.0	9	24.3		
	Not Sure	1	5.3	3	9.1	5	8.3	11	7.3	3	8.1		
Do you prescribe high dose methylprednisolone to patients presenting with acute spinal cord injury with neurological findings?	Always	5	26.3	3	9.1	19	32.2	59	39.6	13	35.1	31.633	**0.002**
	Sometimes	6	31.6	7	21.2	23	39.0	54	36.2	15	40.5		
	Never	7	36.8	21	63.6	14	23.7	32	21.5	9	24.3		
	Not Sure	1	5.3	2	6.1	3	5.1	4	2.7	0	0.0		
Do you perform fusion in patients treated for lumbar stenosis with or without spondylolisthesis after decompression?	Always	4	21.1	6	18.2	4	6.7	36	24.0	4	10.8	27.836	**0.006**
	Sometimes	12	63.2	27	81.8	44	73.3	92	61.3	30	81.1		
	Never	3	15.8	0	0.0	6	10.0	19	12.7	2	5.4		
	Not Sure	0	0.0	0	0.0	6	10.0	3	2.0	1	2.7		
Which of the following statements about the surgical treatment of cervical radiculopathy do you agree with the most?	When correcting cervical sagittal alignment. ACDF alone is sufficient.	4	21.1	15	45.5	17	28.3	48	32.0	16	43.2	21.132	**0.048**
	Anterior surgery results in better outcomes than posterior surgery	4	21.1	10	30.3	18	30.0	49	32.7	7	18.9		
	TDA results in better outcomes than ACDF when treating single level soft disc herniations.	4	21.1	0	0.0	8	13.3	19	12.7	10	27.0		
	The Neck Disability Index. SF-36, SF-12 and VAS are recommended outcome measures	7	36.8	8	24.2	17	28.3	34	22.7	4	10.8		
What is your recommendation to a patient with unilateral extruded disc herniation with no leg pain at the time of your examination?	Microdiscectomy	6	31.6	3	9.1	6	10.0	29	19.3	3	8.1	15.030	0.240
	Physical therapy	5	26.3	13	39.4	19	31.7	44	29.3	12	32.4		
	Medical therapy	4	21.1	9	27.3	27	45.0	54	36.0	17	45.9		
	No treatment	4	21.1	8	24.2	8	13.3	23	15.3	5	13.5		

#### Clinical practice setting

Participants were asked to choose academic practice, hospital employment, private practice, or other categories. However, only a few statistically significant differences were found between participants’ responses in different clinical practice settings. In a few instances, a significant difference was observed among participants working in different clinical practice settings regarding validity and randomization. These include a higher trust in Opinion/Commentary (F = 4.162, *p* = 0.007) and Case Reports (F = 3.163, *p* = 0.025) among privately practicing physicians. Although Systematic Review and Meta-Analysis of Randomized Controlled Trials was the highest in terms of validity in all groups, yet again, privately practicing physicians had the lowest score with an average of 4.20 (F = 3.028, *p* = 0.030). Different practice settings have a certain amount of variability in their approaches to spinal pathologies. For instance, a specific guideline or scoring system in the decision-making process for spinal pathologies is generally followed in all clinical practice settings. ‘Always’ or ‘frequently’ options are chosen primarily in every clinic setting, indicating that there is usually adherence to a certain standard in decision processes. In cases where a guideline or scoring system is not used for spinal pathologies, the rates of making decisions based on face-to-face consultation (X2 = 10.631, *p* = 0.014) or literature search (X2 = 8.888, *p* = 0.031) are higher in the hospital environment. Overall, there is some variability in approaches to spinal pathologies depending on clinical practice settings, but it is seen that the general trends are mainly similar.

When comparing clinical practice settings there were no observed statistically significant differences amongst respondents on the assessment understanding of EBM (Online resource 1: Table 8). However, the percentage of spinal surgeons who were affiliated with an academic institution responded less frequently that EBM is a combination evidence from scientific literature and personal experience, compared with the percentage of spinal surgeons from hospital or private practice (Online resource 1: Table 8). The belief in the need for training on research and methodology in spine surgery was highest amongst spinal surgeons who affiliated with an academic institution (93%) compared with hospital (86.1%) and private practice (75.6%) (Online resource 1: Table 8).

Guidelines or scoring systems in the decision-making process for spinal pathologies were generally followed in all clinical practice settings. “Always” or “frequently” options were chosen mostly for every clinic setting, indicating that there is usually adherence to a certain standard in decision processes (Online resource 1: Table 15). In cases where a guideline or scoring system is not used for spinal pathologies, decisions are mostly based on personal experience. However, while the rates of making decisions based on face-to-face consultation or literature search are higher in the hospital environment, it is seen that private practice and the ‘Other’ category rely more on personal experience. Regarding patients with severe low back pain with no obvious red signs, it is seen that the first step of the clinics is usually a radiological examination (X-ray/CT or MRI) or prescribing medication. This applies to almost every clinical practice setting in most cases. Overall, there is some variability in approaches to spinal pathologies depending on clinical practice settings, but it is seen that the general trends are largely similar.

#### Years of medical practice

The participants were divided into five groups based on years of medical practice (Currently Training, 0–5, 6–10, 11–15, > 15 years). Cross-table analysis showed that there was no statistically significant difference in the participants’ understanding of EBM, their definitions, and their understanding of various concepts related to it, such as validity, randomization, and double-blind, based on the years of medical practice (*p* > 0.05 for all cases). The majority of participants (64–73.2%) have stated that they understand EBM and use it in their practice, and this rate has stayed chiefly the same according to the years of medical practice. Also, most participants (64–75.7%) have indicated that the best definition of “evidence-based medicine” is “combining personal clinical experience with the best evidence derived from medical, scientific research,” and again, this rate has not significantly changed according to the years of medical practice.

A chi-square test was performed for each category’s decisions in various spinal pathologies. In the question “In patients where no guideline or scoring system is used, what is your decision mostly based on?“, “Social media collaboration with colleagues” showed significant variance with a *p*-value below 0.001. Currently Training and the 11–15 years of experience group had a higher rate of social media collaboration. It can be interesting to note that more experienced practitioners (15 + years of medical practice) frequently adhere to guidelines or scoring systems and more regularly base their decisions on personal experience rather than on social media collaboration.

Similarly, for the question “In a patient applying to your clinic with severe low back pain that has affected their quality of life with no red-flag signs detected during examination, what is your FIRST step?“, the responses varied significantly with a *p*-value of 0.026. The Currently Training group opted for X-ray/CT, whereas the advanced years of medical practice groups tended to obtain MRI. “Do you believe the choice of surgical approach improves clinical outcomes in patients with thoracic and lumbar fractures?” also showed significant differences among the responses with a *p*-value of 0.036. While the training group mostly responded as Always, with advancing years, this trend shifted towards Sometimes. In other questions, no significant variation was observed despite differences in percentage values across different years of practice.

#### Additional training for spine surgery

The participants were asked if they received additional training/education/ fellowship/ subspecialty for Spine surgery. In general, the differences in applying EBM between the participants who have and have not received training/education are not statistically significant (*p* > 0.05). However, almost a third (30.8% of those who received training, 27.8% of those who did not) indicated they do not have an idea about “Validity.”

There was no statistically significant difference in the opinions on the validity of study designs and the effectiveness of randomization methods between doctors who have received additional training/education/fellowship/subspecialty for spine surgery and those who have not. A significant difference between clinicians who always adhere to guidelines or scoring systems in spinal pathologies is 51.3% for those who have received additional training in spine surgery and 40.3% for those who have not (*p* = 0.020). This significant difference reflects potential continuation of EBM practices.

In a patient presenting to the clinic with severe low back pain, 42.5% of clinicians who have received additional training in spine surgery prefer to obtain an MRI as the first step, while this preference is at 32.2% among those who have not. However, this difference is not statistically significant (*p* = 0.251). The proportion of those who believe that adding arthrodesis to instrumented fixation improves outcomes in patients with thoracic and lumbar burst fractures is 33.3% in the spine surgery-trained group and 28.3% in the untrained group (*p* = 0.069). The proportion of those who prescribe high-dose methylprednisolone to patients presenting with acute spinal cord injury with neurological findings is 29.4% in the spine surgery-trained group and 36.3% in the untrained group (*p* = 0.352). The proportion of those who recommended microdiscectomy to a patient with unilateral extruded disc herniation with no leg pain at the time of the examination was 9.2% in the group trained in spine surgery and 20% in the untrained group (*p* = 0.004).

## Discussion

From a surgical perspective, assessing the implementation and knowledge of up-to-date EBM findings is difficult. Each surgeon has a different approach tailored to suit the patient’s needs, aiming for maximal benefit. Thus, no clear method exists to validate a surgeon’s decision regarding EBM. This study aimed to shed light on this matter with questions based on the evidence-based clinical guidelines proposed by the North American Spine Society and Congress of Neurological Surgeons. Precisely 300 participants completed the survey, which is comparable to other international surveys conducted in spinal surgery [15–17].

While some learning differences were observed between age groups in the survey, learning trends were generally similar across all respondents’ ages. These learning trends may be accounted for by incorporating EBM in spine surgery education, with or without the label. The statistical differences in age groups for approaches may be attributed to the years of experience and network of colleagues available to provide alternative solutions on a case-by-case basis.

One concern supported by this survey is how EBM is perceived across geographical regions. As noted in the Results, spinal surgeons from Asia and Africa answered less accurately on EBM’s correct definition and use than those of North America and Europe. Since EBM can be successfully applied in spine surgery [2, 18–21], future training and education must incorporate the teaching of proper EBM understanding and implementation. The incorporation of EBM in education and training is supported by current literature [22]. Furthermore, it may be worth it for spinal surgeons to use social media [7], conferences [24], or training online to integrate EBM education opportunities to more easily reach underdeveloped regions of the world and provide opportunities for those who were unable to acquire this education during their medical schooling.

Low back pain is one of the most common reasons for applying to a spinal surgeon. However, the order of obtaining further diagnostic imaging modalities in the absence of red flags remains a debate. Most of the participants opted to obtain an MRI. However, there is insufficient evidence to recommend for or against obtaining imaging in the absence of red flags and the type of imaging [25]. A striking difference was observed in the imaging modality amongst geographical locations. While most participants from North America and Europe opted for an MRI, most respondents from Asia and Africa stated they’d prefer an X-ray/CT before an MRI. This may be due to a higher availability of X-ray/CT compared to MRI in regions from responders who were less likely to order an MRI [26–28]; alternatively, the recommendation for X-ray/CT instead of MRI could be if a patient does not present with any red flags with lower back pain that warrants an MRI. Thus, X-ray/CT is chosen first. Another clinical scenario evaluated was an extruded disc with no leg pain. These patients sometimes undergo unnecessary surgery due to radiological findings; however, most of the respondents opted for physical and medical therapy. The notion of “treat the patient, not the image” has been well settled in the spine surgery society. However, the debate of “what to treat with physical and medical therapy if no leg pain is present” arises at this point.

Our results indicated differing trust in commentary per clinical setting. Those who operated private practice were significantly more likely to trust opinion/ commentary and case reports. While some case reports within spine surgery can offer insight into procedural and surgical nuance, the trust in opinion and commentary as opposed to RCT or systematic reviews and meta-analyses is a surprising rift between other clinical settings. These differences could be due to the change in environment; perhaps not being in a hospital or institution setting offers less inclination to utilize other research than trusted resources. Therefore, the environment in which a surgeon practices spinal surgery could significantly affect how the surgeon stays updated on surgical recommendations and influence what a surgeon prefers for insight into patient treatment options.

Interestingly, those who received fellowships/additional training in spine surgery did not differ much in responses when compared to those who did not receive additional training in spine surgery. These findings suggest that medical professionals widely accept the concept of EBM. However, we did not expect about a third of the respondents with additional training to indicate that they did not know what validity meant. This lack of familiarity reinforces the need for additional training in spine surgery education to incorporate teaching familiarization of proper research interpretation, such as explaining validity, randomization, and blinding, since these facets lay the foundations for EBM and avoid the misuse of it. Yet there was a 9.2 to 20% ratio between the additional training and no additional training groups of those who recommended and those who did not recommend microdiscectomy to a patient with unilateral extruded disc herniation with no leg pain at the time of the examination. Thus, this data illuminates the role of spine surgery training in determining clinicians’ approaches to spinal pathologies and treatment options; it also demonstrates how this training can affect the clinical decision-making process in certain situations.

When treating thoracolumbar fractures, most participants stated that the surgical approach site and the addition of arthrodesis improves clinical outcomes. However, there is sufficient data on the insignificance of the site of approach with regards to improved clinical outcome (Grade B) and that the addition of arthrodesis to instrumentation has not been shown to impact outcomes and increases blood loss and operative time (Grade A) [18]. In this manner, the surgeon’s personal experience and the “main-stray” approach in thoracolumbar fractures may have affected these decisions. However, we see that implementing EBM in thoracolumbar fractures is necessary and should be focused on.

Another conundrum in spinal surgery is whether low BMD should be treated before spinal fusion surgeries. As the number of instrumentation requiring spinal surgeries are increasing, so are the number of failed fusion and screw loosening cases [29]. Although there were different trends towards the treatment before surgery, most participants responded that they always or sometimes treat BMD. There is a grade B strength of recommendation for the use of teriparatide in patients with osteoporosis, but insufficient data on the use of bisphosphonates to decrease postoperative adverse events [21]. We believe the spinal surgical society is well aware of the adverse events of fusion surgery and its relation to low BMD. However, the choice and duration of treatment, along with the cut-off BMD value for treatment, are areas that need further investigation.

The employment of high-dose steroids after spinal cord injury (SCI) is also a popular debate topic amongst spine surgeons. Although both the CNS and AANS published results against the use of methylprednisolone for patients with acute SCI and adverse events, including mortality, exist [30], many of the respondents recommended the use of steroids. More unexpectedly, when considering those who received further training in spine surgery, a third of these respondents prescribed methylprednisolone for patients with acute SCI. Regarding the treatment of cervical radiculopathy, most participants agreed that ACDF alone is a sufficient treatment of sagittal alignment [4].

The high rate of always or frequently referring to guidelines or scoring systems during decisions signifies the importance of adherence to current medical knowledge and evidence-based medical practices. Additionally, the likelihood for participants to consider decisions through a literature search or by personal experience without guidelines or scoring systems indicates that experience and scientific literature play a substantial role in clinical decision-making. Furthermore, this suggests a trend towards employing EBM in practice more often instead of relying firstly on personal experience for the aforementioned clinical scenario since most of the responders were surveying scientific literature before deciding. However, the authors expected the frequency of choosing face-to-face consultations in this scenario to be similar to personal experience and literary search since first-hand experience from a colleague can sometimes be more readily available information than a literature search. Thus, the preference to survey the literature more often than relying on personal experience or face-to-face consultation could suggest that most surgeons in spine surgery have enough familiarity to comb scientific literature for decision-making timely; thus, recent adjustments in medical training and education to improve navigation of medical databases may account for the high frequency of this choice.

Recent years have shown EBM internationally emerging as a trustworthy standard for clinical practice [31–34], the surgical side of medicine has been reportedly hesitant to adopt EBM [19, 35] due to the individuality of patients and their cases, and the scale of skill that changes depending on the surgeon. Furthermore, there are numerous cases without high-level evidence for any specific direction in treatment. This survey suggests that surgeons encountering cases like these are willing to delve into literature and collaborate with colleagues through social media, face-to-face consultations, or personal experience. The survey doesn’t directly answer whether or not surgeons in spinal surgery believe these methods to be contrary to EBM, but it sheds some light. A majority of the participants understood that EBM included using the most updated and validated literature for treatments in addition to clinical experience. In the cases where no substantial evidence or recommendation exists, the personal experience of the surgeon and surgical colleagues is within the confines of EBM. Since most respondents understand this, it may indicate that the greater spinal surgery community understands their use of EBM includes their surgical experience especially when no evidence for treatment of a case is available. However, it is challenging to implement a precise algorithm based entirely on EBM and expect all spine surgeons to follow through with it. Practice guidelines created by different spine-related societies may be helpful for general spine practitioners. Some examples include NASS, AO-Spine, AANS/CNS, and WFNS Spine Committee guidelines. The diagnostic and treatment modalities are decided per patient, disease, and surgeon. However, these guidelines certainly shape and affect surgeons’ decision-making perspectives.

### Limitations

One of the core limitations of this study is the imbalance of participants from geographical regions and the number of women participants. It is important to have a balance of participants from geographical areas to gauge the perspectives and responses by region adequately. The percentage of women participants was similar to representation by percentage in orthopedic surgery and neurosurgery [20, 36–38]. However, the number of respondents was too low to assess survey responses between gender groups confidently. Future surveys should utilize organizations for women physicians, such as the Women in Neurosurgery organization, to increase the number of women respondents. Another potential limitation of this survey is the need for insight into how the COVID pandemic has influenced the application of and learning of EBM in spinal surgery since there have been many indirect consequences to learning medical education attributed to the pandemic [40, 41]. On the topic of education, it would’ve been insightful to know what range of EBM education the participants had, whether formal or informal. Comparing responses regarding the correct understanding of EBM with the level of EBM education received could have resulted in insightful information regarding the education on the application gap of EBM in spinal surgery.

## Conclusions

This study revealed that most respondents used EBM in practice and that nearly all believed training in research methodology and EBM is necessary for the practice of spine surgery. However, there are disagreements on how EBM should be applied per case. Significant differences in reasoning were discovered based on geographical region, advanced training, practice setting, and other factors. These results illustrate EBM’s limitations. Thus, the appropriate application of EBM in clinical settings for spinal surgery must be further studied.

## Electronic supplementary material

Below is the link to the electronic supplementary material.

Supplementary Material 1

## Data Availability

The full comprehensive data set collected during this study is provided through the supplementary files.
