# Supplementary material for: Do we really apply evidence-based-recommendations to spine surgery? Results of an international survey
Source: Neurosurg Rev. 2024 Jun 10;47(1):264. doi: 10.1007/s10143-024-02502-0 (PMC11164786; doi:10.1007/s10143-024-02502-0)
Supplement: Supplementary file 1 — Supplementary Material 1 [file 10143_2024_2502_MOESM1_ESM.docx]

**Complete Data Set**

**Table 1. Characteristics of the Responders to the Survey**

|  |  | **n** | **%** |
| --- | --- | --- | --- |
| Gender | Male | 264 | 88.0 |
|  | Female | 36 | 12.0 |
| Age (years) | ≤30 | 39 | 13.0 |
|  | 31-40 | 138 | 46.0 |
|  | 41-50 | 82 | 27.3 |
|  | 51-60 | 31 | 10.3 |
|  | 61-70 | 9 | 3.0 |
|  | >70 | 1 | 0.3 |
| Region of practice | North America | 19 | 6.3 |
|  | South America | 33 | 11.0 |
|  | Europe | 60 | 20.0 |
|  | Asia | 150 | 50.0 |
|  | Oceania | 1 | 0.3 |
|  | Africa | 37 | 12.3 |
| Specialty | No | 2 | 0.7 |
|  | Neurosurgeon | 287 | 95.7 |
|  | Orthopedics | 7 | 2.3 |
|  | Spine | 0 | 0.0 |
|  | Other | 4 | 1.3 |
| Status | Resident | 84 | 28.0 |
|  | Consultant | 145 | 48.3 |
|  | Academic | 52 | 17.3 |
|  | Other | 19 | 6.3 |
| Clinical Practice Setting | Academic Practice | 86 | 28.7 |
|  | Hospital Employment | 166 | 55.3 |
|  | Private Practice | 41 | 13.7 |
|  | Military | 2 | 0.7 |
|  | Other | 5 | 1.7 |
| Years of Medical Practice (total) | Currently Training | 33 | 11.0 |
|  | 0-5 | 74 | 24.7 |
|  | 6-10 | 56 | 18.7 |
|  | 11-15 | 62 | 20.7 |
|  | 16-20 | 30 | 10.0 |
|  | >20 | 45 | 15.0 |
| Have you received additional training/education/fellowship/subspecialty for Spine Surgery? | Yes | 120 | 40.0 |
|  | No | 180 | 60.0 |

**Table 2. Most Frequent Responses in the Assessment of EBM**

|  |  | **n** | **%** |
| --- | --- | --- | --- |
| Are you familiar with the concept “evidence-based medicine” (EBM)? | I know and understand EBM but do not use it | 64 | 21.3 |
|  | I know and understand EBM and use it during my practice | 203 | 67.7 |
|  | I have heard of it but don’t understand what it is | 27 | 9.0 |
|  | I have never heard of it | 6 | 2.0 |
| Which of the following definitions do you think BEST defines “evidence-based medicine”? | Exclusive use of personal clinical experience of the practitioner in medical decision. | 19 | 6.3 |
|  | Exclusive use of medical research results in medical decision. | 60 | 20.0 |
|  | Combining personal clinical experience with the best evidence derived from medical scientific... | 213 | 71.0 |
|  | I do not know | 8 | 2.7 |
| Which of the following statements is FALSE concerning “VALIDITY”? | Validity includes two domains: internal and external validity. | 27 | 9.0 |
|  | Validity refers to how well the results represent true findings outside the study. | 80 | 26.7 |
|  | Internal and external validity can be both performed independent of each other. | 72 | 24.0 |
|  | A low external validity reduces the generalizability of the study findings. | 34 | 11.3 |
|  | I do not know | 87 | 29.0 |
| How would you define RANDOMIZATION? | Treatment and control patients are followed an equal amount of time and treated the same | 73 | 24.3 |
|  | Outcomes between treatment and control groups are the same | 19 | 6.3 |
|  | Both known and unknown prognostic factors are equally balanced between treatment and control patient groups | 199 | 66.3 |
|  | Patients receive the treatment or control that surgeons’ prefer the most | 7 | 2.3 |
|  | Never heard of it | 2 | 0.7 |
| In a randomized controlled study, how would you define double-blind? | Patients and outcome assessors are blind | 62 | 20.7 |
|  | Researcher and outcome assessors are blind | 56 | 18.7 |
|  | Patients and researchers are blind | 100 | 33.3 |
|  | Patients, researchers and outcome assessors are blind | 65 | 21.7 |
|  | I don’t have an idea | 17 | 5.7 |
| Do you believe a training for research methodology and evidence based medicine is necessary in neurosurgery? | Yes | 258 | 86.0 |
|  | Partially | 34 | 11.3 |
|  | No | 7 | 2.3 |
|  | Not Sure | 1 | 0.3 |
| Do you take into consideration the “level of evidence” or “grade of recommendation” provided by the study when you are reading a published scientific article? | Always | 106 | 35.3 |
|  | Frequently | 167 | 55.7 |
|  | Rarely | 25 | 8.3 |
|  | Never | 2 | 0.7 |
| Evidence-based medicine is used to.. | Make medical decisions correctly and scientifically for the patient’s benefit | 246 | 82.0 |
|  | Know the correct form of a manuscript | 20 | 6.7 |
|  | Learn what's new in the medical field | 18 | 6.0 |
|  | Disregard of evidence-based medicine in any individual study or current practice | 10 | 3.3 |
|  | I have not yet understood the usefulness of evidence-based medicine | 6 | 2.0 |

**Table 3. Most Frequent Answers to Various Clinical Scenarios**

|  |  | **n** | **%** |
| --- | --- | --- | --- |
| Do you adhere to any guidelines or scoring systems when making decisions in spinal pathologies? | Always | 132 | 44.0 |
|  | Frequently | 151 | 50.3 |
|  | Rarely | 12 | 4.0 |
|  | Never | 5 | 1.7 |
| In patients where no guideline or scoring system is used, what is your decision mostly based on? | Personal experience | 148 | 49.3 |
|  | Face-to-face consultation with neurosurgeons | 133 | 44.3 |
|  | Social media collaboration with neurosurgeons | 41 | 13.7 |
|  | Literature search | 165 | 55.0 |
|  | Reference text-books | 98 | 32.7 |
|  | Other | 4 | 1.3 |
| In a patient applying to your clinic with severe low back pain that has affected their quality of life with no red-flag signs (radiculopathy, bladder problems, deficits etc.) detected during examination, what is your FIRST step? | Obtain X-ray/CT | 69 | 23.0 |
|  | Obtain MRI | 109 | 36.3 |
|  | Prescribe medication | 93 | 31.0 |
|  | Suggest bed-rest | 16 | 5.3 |
|  | None | 13 | 4.3 |
| Do you believe the choice of surgical approach (anterior, posterior, or combined anterior posterior) improve clinical outcomes in patients with thoracic and lumbar fractures? | Always | 116 | 38.7 |
|  | Sometimes | 163 | 54.3 |
|  | Never | 9 | 3.0 |
|  | Not Sure | 12 | 4.0 |
| Do you believe the addition of arthrodesis to instrumented fixation improve outcomes in patients with thoracic and lumbar burst fractures? | Always | 91 | 30.3 |
|  | Sometimes | 176 | 58.7 |
|  | Never | 11 | 3.7 |
|  | Not Sure | 22 | 7.3 |
| Do you treat low BMD (Bone Mineral Density T <-2.5) before spine surgery? | Always | 83 | 27.7 |
|  | Sometimes | 158 | 52.7 |
|  | Never | 36 | 12.0 |
|  | Not Sure | 23 | 7.7 |
| Do you prescribe high dose methylprednisolone to patients presenting with acute spinal cord injury with neurological findings? | Always | 100 | 33.6 |
|  | Sometimes | 105 | 35.2 |
|  | Never | 83 | 27.9 |
|  | Not Sure | 10 | 3.4 |
| Do you perform fusion in patients treated for lumbar stenosis with or without spondylolisthesis after decompression? | Always | 54 | 18.0 |
|  | Sometimes | 205 | 68.3 |
|  | Never | 31 | 10.3 |
|  | Not Sure | 10 | 3.3 |
| Which of the following statements about the surgical treatment of cervical radiculopathy do you agree with the most? | When correcting cervical sagittal alignment, ACDF alone is sufficient. | 100 | 33.3 |
|  | Anterior surgery results in better outcomes than posterior surgery | 89 | 29.7 |
|  | TDA results in better outcomes than ACDF when treating single level soft disc herniations. | 41 | 13.7 |
|  | The Neck Disability Index, SF-36, SF-12 and VAS are recommended outcome measures for assessing ... | 70 | 23.3 |
| What is your recommendation to a patient with unilateral extruded disc herniation with no leg pain at the time of your examination? | Microdiscectomy | 47 | 15.7 |
|  | Physical therapy | 93 | 31.0 |
|  | Medical therapy | 112 | 37.3 |
|  | No treatment | 48 | 16.0 |

**Table 4. Comparison of Age Groups in Various Clinical Scenarios**

|  | | **Age (years)** | | | | | | | | **X^2^** | **p** |
| --- | --- | --- | --- | --- | --- | --- | --- | --- | --- | --- | --- |
|  |  | **≤30** | | **31-40** | | **41-50** | | **50>** | |  |  |
|  |  | **n** | **%** | **n** | **%** | **n** | **%** | **n** | **%** |  |  |
| Are you familiar with the concept “evidence-based medicine” (EBM)? | I know and understand EBM but do not use it | 7 | 17.9 | 26 | 18.8 | 17 | 20.7 | 14 | 34.1 | 8.459 | 0.489 |
|  | I know and understand EBM and use it during my practice | 27 | 69.2 | 93 | 67.4 | 59 | 72.0 | 24 | 58.5 |  |  |
|  | I have heard of it but don’t understand what it is | 4 | 10.3 | 15 | 10.9 | 6 | 7.3 | 2 | 4.9 |  |  |
|  | I have never heard of it | 1 | 2.6 | 4 | 2.9 | 0 | 0.0 | 1 | 2.4 |  |  |
| Which of the following definitions do you think BEST defines “evidence-based medicine”? | Exclusive use of personal clinical experience of the practitioner in medical decision. | 4 | 10.3 | 7 | 5.1 | 5 | 6.1 | 3 | 7.3 | 9.370 | 0.404 |
|  | Exclusive use of medical research results in medical decision. | 6 | 15.4 | 25 | 18.1 | 21 | 25.6 | 8 | 19.5 |  |  |
|  | Combining personal clinical experience with the best evidence derived from medical scientific... | 29 | 74.4 | 99 | 71.7 | 55 | 67.1 | 30 | 73.2 |  |  |
|  | I do not know | 0 | 0.0 | 7 | 5.1 | 1 | 1.2 | 0 | 0.0 |  |  |
| Which of the following statements is FALSE concerning “VALIDITY”? | Validity includes two domains: internal and external validity. | 4 | 10.3 | 12 | 8.7 | 5 | 6.1 | 6 | 14.6 | 5.109 | 0.954 |
|  | Validity refers to how well the results represent true findings outside the study. | 11 | 28.2 | 37 | 26.8 | 22 | 26.8 | 10 | 24.4 |  |  |
|  | Internal and external validity can be both performed independent of each other. | 11 | 28.2 | 33 | 23.9 | 21 | 25.6 | 7 | 17.1 |  |  |
|  | A low external validity reduces the generalizability of the study findings. | 5 | 12.8 | 14 | 10.1 | 10 | 12.2 | 5 | 12.2 |  |  |
|  | I do not know | 8 | 20.5 | 42 | 30.4 | 24 | 29.3 | 13 | 31.7 |  |  |
| How would you define RANDOMIZATION? | Treatment and control patients are followed an equal amount of time and treated the same | 8 | 20.5 | 29 | 21.2 | 23 | 28.0 | 13 | 32.5 | 5.111 | 0.825 |
|  | Outcomes between treatment and control groups are the same | 1 | 2.6 | 10 | 7.3 | 6 | 7.3 | 2 | 5.0 |  |  |
|  | Both known and unknown prognostic factors are equally balanced between treatment and control patient groups | 29 | 74.4 | 94 | 68.6 | 52 | 63.4 | 24 | 60.0 |  |  |
|  | Patients receive the treatment or control that surgeons’ prefer the most | 1 | 2.6 | 4 | 2.9 | 1 | 1.2 | 1 | 2.5 |  |  |
| In a randomized controlled study, how would you define double-blind? | Patients and outcome assessors are blind | 9 | 23.1 | 33 | 23.9 | 12 | 14.6 | 8 | 19.5 | 13.077 | 0.363 |
|  | Researcher and outcome assessors are blind | 8 | 20.5 | 20 | 14.5 | 22 | 26.8 | 6 | 14.6 |  |  |
|  | Patients and researchers are blind | 11 | 28.2 | 44 | 31.9 | 33 | 40.2 | 12 | 29.3 |  |  |
|  | Patients, researchers and outcome assessors are blind | 8 | 20.5 | 33 | 23.9 | 12 | 14.6 | 12 | 29.3 |  |  |
|  | I don’t have an idea | 3 | 7.7 | 8 | 5.8 | 3 | 3.7 | 3 | 7.3 |  |  |
| Do you believe a training for research methodology and evidence based medicine is necessary in neurosurgery? | Yes | 34 | 87.2 | 123 | 89.1 | 71 | 87.7 | 30 | 73.2 | 8.182 | 0.225 |
|  | Partially | 4 | 10.3 | 12 | 8.7 | 8 | 9.9 | 10 | 24.4 |  |  |
|  | No | 1 | 2.6 | 3 | 2.2 | 2 | 2.5 | 1 | 2.4 |  |  |
| Do you take into consideration the “level of evidence” or “grade of recommendation” provided by the study when you are reading a published scientific article? | Always | 18 | 46.2 | 47 | 34.6 | 30 | 36.6 | 11 | 26.8 | 9.111 | 0.167 |
|  | Frequently | 20 | 51.3 | 76 | 55.9 | 48 | 58.5 | 23 | 56.1 |  |  |
|  | Rarely | 1 | 2.6 | 13 | 9.6 | 4 | 4.9 | 7 | 17.1 |  |  |
| Evidence-based medicine is used to.. | Make medical decisions correctly and scientifically for the patient’s benefit | 33 | 84.6 | 109 | 79.0 | 70 | 85.4 | 34 | 82.9 | x | x |
|  | Know the correct form of a manuscript | 3 | 7.7 | 10 | 7.2 | 4 | 4.9 | 3 | 7.3 |  |  |
|  | Learn what's new in the medical field | 1 | 2.6 | 8 | 5.8 | 6 | 7.3 | 3 | 7.3 |  |  |
|  | Disregard of evidence-based medicine in any individual study or current practice | 2 | 5.1 | 8 | 5.8 | 0 | 0.0 | 0 | 0.0 |  |  |
|  | I have not yet understood the usefulness of evidence-based medicine | 0 | 0.0 | 3 | 2.2 | 2 | 2.4 | 1 | 2.4 |  |  |

**Table 5. Comparison of Region of Practice in Various Clinical Scenarios**

|  | | **Region of practice** | | | | | | | | | | **X^2^** | **p** |
| --- | --- | --- | --- | --- | --- | --- | --- | --- | --- | --- | --- | --- | --- |
|  |  | **North America** | | **South America** | | **Europe** | | **Asia** | | **Africa** | |  |  |
|  |  | **n** | **%** | **n** | **%** | **n** | **%** | **n** | **%** | **n** | **%** |  |  |
| Do you adhere to any guidelines or scoring systems when making decisions in spinal pathologies? | Always | 8 | 44.4 | 9 | 27.3 | 23 | 39.0 | 74 | 50.0 | 18 | 50.0 | 12.004 | 0.151 |
|  | Frequently | 10 | 55.6 | 23 | 69.7 | 31 | 52.5 | 68 | 45.9 | 18 | 50.0 |  |  |
|  | Rarely | 0 | 0.0 | 1 | 3.0 | 5 | 8.5 | 6 | 4.1 | 0 | 0.0 |  |  |
| In patients where no guideline or scoring system is used, what is your decision mostly based on? | Personal experience | 12 | 63.2 | 11 | 33.3 | 30 | 50.0 | 73 | 48.7 | 22 | 59.5 | 6.384 | 0.172 |
|  | Face-to-face consultation with neurosurgeons | 10 | 52.6 | 16 | 48.5 | 37 | 61.7 | 53 | 35.3 | 16 | 43.2 | 13.013 | **0.011** |
|  | Social media collaboration with neurosurgeons | 1 | 5.3 | 1 | 3.0 | 6 | 10.0 | 26 | 17.3 | 7 | 18.9 | 7.538 | 0.110 |
|  | Literature search | 14 | 73.7 | 18 | 54.5 | 47 | 78.3 | 68 | 45.3 | 18 | 48.6 | 22.16 | **0.000** |
|  | Reference text-books | 8 | 42.1 | 6 | 18.2 | 20 | 33.3 | 52 | 34.7 | 12 | 32.4 | 4.194 | 0.380 |
| In a patient applying to your clinic with severe low back pain that has affected their quality of life with no red-flag signs (radiculopathy, bladder problems, deficits etc.) detected during examination, what is your FIRST step? | Obtain X-ray/CT | 4 | 21.1 | 6 | 18.2 | 5 | 8.3 | 41 | 27.3 | 12 | 32.4 | 34.295 | **0.005** |
|  | Obtain MRI | 8 | 42.1 | 10 | 30.3 | 30 | 50.0 | 53 | 35.3 | 8 | 21.6 |  |  |
|  | Prescribe medication | 5 | 26.3 | 13 | 39.4 | 21 | 35.0 | 39 | 26.0 | 15 | 40.5 |  |  |
|  | Suggest bed-rest | 0 | 0.0 | 0 | 0.0 | 1 | 1.7 | 13 | 8.7 | 2 | 5.4 |  |  |
|  | None | 2 | 10.5 | 4 | 12.1 | 3 | 5.0 | 4 | 2.7 | 0 | 0.0 |  |  |
| Do you believe the choice of surgical approach (anterior, posterior, or combined anterior posterior) improve clinical outcomes in patients with thoracic and lumbar fractures? | Always | 8 | 42.1 | 10 | 30.3 | 16 | 26.7 | 67 | 44.7 | 15 | 40.5 | 15.479 | 0.216 |
|  | Sometimes | 11 | 57.9 | 21 | 63.6 | 41 | 68.3 | 72 | 48.0 | 18 | 48.6 |  |  |
|  | Never | 0 | 0.0 | 2 | 6.1 | 0 | 0.0 | 4 | 2.7 | 2 | 5.4 |  |  |
|  | Not Sure | 0 | 0.0 | 0 | 0.0 | 3 | 5.0 | 7 | 4.7 | 2 | 5.4 |  |  |
| Do you believe the addition of arthrodesis to instrumented fixation improve outcomes in patients with thoracic and lumbar burst fractures? | Always | 8 | 42.1 | 10 | 30.3 | 8 | 13.3 | 53 | 35.3 | 11 | 29.7 | 20.456 | **0.059** |
|  | Sometimes | 9 | 47.4 | 23 | 69.7 | 44 | 73.3 | 79 | 52.7 | 21 | 56.8 |  |  |
|  | Never | 1 | 5.3 | 0 | 0.0 | 1 | 1.7 | 6 | 4.0 | 3 | 8.1 |  |  |
|  | Not Sure | 1 | 5.3 | 0 | 0.0 | 7 | 11.7 | 12 | 8.0 | 2 | 5.4 |  |  |
| Do you treat low BMD (Bone Mineral Density T <-2.5) before spine surgery? | Always | 3 | 15.8 | 17 | 51.5 | 5 | 8.3 | 49 | 32.7 | 9 | 24.3 | 34.590 | **0.001** |
|  | Sometimes | 14 | 73.7 | 9 | 27.3 | 40 | 66.7 | 78 | 52.0 | 16 | 43.2 |  |  |
|  | Never | 1 | 5.3 | 4 | 12.1 | 10 | 16.7 | 12 | 8.0 | 9 | 24.3 |  |  |
|  | Not Sure | 1 | 5.3 | 3 | 9.1 | 5 | 8.3 | 11 | 7.3 | 3 | 8.1 |  |  |
| Do you prescribe high dose methylprednisolone to patients presenting with acute spinal cord injury with neurological findings? | Always | 5 | 26.3 | 3 | 9.1 | 19 | 32.2 | 59 | 39.6 | 13 | 35.1 | 31.633 | **0.002** |
|  | Sometimes | 6 | 31.6 | 7 | 21.2 | 23 | 39.0 | 54 | 36.2 | 15 | 40.5 |  |  |
|  | Never | 7 | 36.8 | 21 | 63.6 | 14 | 23.7 | 32 | 21.5 | 9 | 24.3 |  |  |
|  | Not Sure | 1 | 5.3 | 2 | 6.1 | 3 | 5.1 | 4 | 2.7 | 0 | 0.0 |  |  |
| Do you perform fusion in patients treated for lumbar stenosis with or without spondylolisthesis after decompression? | Always | 4 | 21.1 | 6 | 18.2 | 4 | 6.7 | 36 | 24.0 | 4 | 10.8 | 27.836 | **0.006** |
|  | Sometimes | 12 | 63.2 | 27 | 81.8 | 44 | 73.3 | 92 | 61.3 | 30 | 81.1 |  |  |
|  | Never | 3 | 15.8 | 0 | 0.0 | 6 | 10.0 | 19 | 12.7 | 2 | 5.4 |  |  |
|  | Not Sure | 0 | 0.0 | 0 | 0.0 | 6 | 10.0 | 3 | 2.0 | 1 | 2.7 |  |  |
| Which of the following statements about the surgical treatment of cervical radiculopathy do you agree with the most? | When correcting cervical sagittal alignment, ACDF alone is sufficient. | 4 | 21.1 | 15 | 45.5 | 17 | 28.3 | 48 | 32.0 | 16 | 43.2 | 21.132 | **0.048** |
|  | Anterior surgery results in better outcomes than posterior surgery | 4 | 21.1 | 10 | 30.3 | 18 | 30.0 | 49 | 32.7 | 7 | 18.9 |  |  |
|  | TDA results in better outcomes than ACDF when treating single level soft disc herniations. | 4 | 21.1 | 0 | 0.0 | 8 | 13.3 | 19 | 12.7 | 10 | 27.0 |  |  |
|  | The Neck Disability Index, SF-36, SF-12 and VAS are recommended outcome measures for assessing ... | 7 | 36.8 | 8 | 24.2 | 17 | 28.3 | 34 | 22.7 | 4 | 10.8 |  |  |
| What is your recommendation to a patient with unilateral extruded disc herniation with no leg pain at the time of your examination? | Microdiscectomy | 6 | 31.6 | 3 | 9.1 | 6 | 10.0 | 29 | 19.3 | 3 | 8.1 | 15.030 | 0.240 |
|  | Physical therapy | 5 | 26.3 | 13 | 39.4 | 19 | 31.7 | 44 | 29.3 | 12 | 32.4 |  |  |
|  | Medical therapy | 4 | 21.1 | 9 | 27.3 | 27 | 45.0 | 54 | 36.0 | 17 | 45.9 |  |  |
|  | No treatment | 4 | 21.1 | 8 | 24.2 | 8 | 13.3 | 23 | 15.3 | 5 | 13.5 |  |  |

**Table 6. Responses to validity and randomization**

|  | **Definitely Not Trust** | | **Probably Not Trust** | | **Not Sure** | | **Probably Trust** | | **Definitely Trust** | | **Mean** |
| --- | --- | --- | --- | --- | --- | --- | --- | --- | --- | --- | --- |
|  | **n** | **%** | **n** | **%** | **n** | **%** | **n** | **%** | **n** | **%** |  |
| **VALIDITY of the study designs** |  |  |  |  |  |  |  |  |  |  |  |
| Opinion/Commentary | 26 | 8.7 | 39 | 13.0 | 59 | 19.7 | 137 | 45.7 | 39 | 13.0 | 3.41 |
| Case Report | 10 | 3.3 | 32 | 10.7 | 43 | 14.3 | 155 | 51.7 | 60 | 20.0 | 3.74 |
| Experimental Animal Study | 13 | 4.3 | 22 | 7.3 | 67 | 22.3 | 152 | 50.7 | 46 | 15.3 | 3.65 |
| Retrospective Case Series | 2 | 0.7 | 17 | 5.7 | 30 | 10.0 | 170 | 56.7 | 81 | 27.0 | 4.04 |
| Prospective Case Series | 4 | 1.3 | 6 | 2.0 | 17 | 5.7 | 137 | 45.7 | 136 | 45.3 | 4.32 |
| Observational Study | 4 | 1.3 | 7 | 2.3 | 20 | 6.7 | 147 | 49.0 | 122 | 40.7 | 4.25 |
| Randomized Controlled Trial | 3 | 1.0 | 4 | 1.3 | 19 | 6.3 | 67 | 22.3 | 207 | 69.0 | 4.57 |
| Systematic Review and Meta-Analysis of RCT | 4 | 1.3 | 10 | 3.3 | 12 | 4.0 | 68 | 22.7 | 206 | 68.7 | 4.54 |
| **most effective way to achieve RANDOMIZATION** |  |  |  |  |  |  |  |  |  |  |  |
| Randomized computer algorithm | 28 | 9.3 | 39 | 13.0 | 114 | 38.0 | 79 | 26.3 | 40 | 13.3 | 3.21 |
| Use date of birth | 21 | 7.0 | 55 | 18.3 | 97 | 32.3 | 98 | 32.7 | 29 | 9.7 | 3.20 |
| Use alternate days | 12 | 4.0 | 37 | 12.3 | 93 | 31.0 | 120 | 40.0 | 38 | 12.7 | 3.45 |
| Use patient’s ID number | 14 | 4.7 | 30 | 10.0 | 76 | 25.3 | 109 | 36.3 | 71 | 23.7 | 3.64 |
| Coin Toss | 3 | 1.0 | 4 | 1.3 | 21 | 7.0 | 79 | 26.3 | 193 | 64.3 | 4.52 |
| Use Patient’s Preference | 71 | 23.7 | 44 | 14.7 | 100 | 33.3 | 56 | 18.7 | 29 | 9.7 | 2.76 |
| Use Surgeon’s Preference | 72 | 24.0 | 38 | 12.7 | 87 | 29.0 | 68 | 22.7 | 35 | 11.7 | 2.85 |
| Randomization by the researcher | 31 | 10.3 | 21 | 7.0 | 59 | 19.7 | 102 | 34.0 | 87 | 29.0 | 3.64 |
| **most effective technique to conceal randomization** |  |  |  |  |  |  |  |  |  |  |  |
| Call a separate center via telephone to obtain the next patient allocation | 12 | 4.0 | 18 | 6.0 | 74 | 24.7 | 133 | 44.3 | 63 | 21.0 | 3.72 |
| Use opaque envelopes that contain the next treatment allocation | 5 | 1.7 | 17 | 5.7 | 62 | 20.7 | 132 | 44.0 | 84 | 28.0 | 3.91 |
| Only tell the research assistant/study nurse what the next allocation will be | 17 | 5.7 | 38 | 12.7 | 85 | 28.3 | 112 | 37.3 | 48 | 16.0 | 3.45 |
| Post the randomization schedule on a board in the operating room | 29 | 9.7 | 36 | 12.0 | 78 | 26.0 | 114 | 38.0 | 43 | 14.3 | 3.35 |
| Only tell the operating surgeon what the full randomization schedule is | 40 | 13.3 | 36 | 12.0 | 72 | 24.0 | 103 | 34.3 | 49 | 16.3 | 3.28 |

**Table 7. Differences in Responses in the Assessment of EBM between Genders**

|  | | **Gender** | | | | **X^2^** | **p** |
| --- | --- | --- | --- | --- | --- | --- | --- |
|  |  | **Male** | | **Female** | |  |  |
|  |  | **n** | **%** | **n** | **%** |  |  |
| Are you familiar with the concept “evidence-based medicine” (EBM)? | I know and understand EBM but do not use it | 56 | 21.2 | 8 | 22.2 | 0.670 | 0.881 |
|  | I know and understand EBM and use it during my practice | 177 | 67.0 | 26 | 72.2 |  |  |
|  | I have heard of it but don’t understand what it is | 25 | 9.5 | 2 | 5.6 |  |  |
|  | I have never heard of it | 6 | 2.3 | 0 | 0.0 |  |  |
| Which of the following definitions do you think BEST defines “evidence-based medicine”? | Exclusive use of personal clinical experience of the practitioner in medical decision. | 16 | 6.1 | 3 | 8.3 | 4.971 | 0.137 |
|  | Exclusive use of medical research results in medical decision. | 54 | 20.5 | 6 | 16.7 |  |  |
|  | Combining personal clinical experience with the best evidence derived from medical scientific... | 189 | 71.6 | 24 | 66.7 |  |  |
|  | I do not know | 5 | 1.9 | 3 | 8.3 |  |  |
| Which of the following statements is FALSE concerning “VALIDITY”? | Validity includes two domains: internal and external validity. | 20 | 7.6 | 7 | 19.4 | 9.683 | **0.039** |
|  | Validity refers to how well the results represent true findings outside the study. | 66 | 25.0 | 14 | 38.9 |  |  |
|  | Internal and external validity can be both performed independent of each other. | 67 | 25.4 | 5 | 13.9 |  |  |
|  | A low external validity reduces the generalizability of the study findings. | 31 | 11.7 | 3 | 8.3 |  |  |
|  | I do not know | 80 | 30.3 | 7 | 19.4 |  |  |
| How would you define RANDOMIZATION? | Treatment and control patients are followed an equal amount of time and treated the same | 68 | 26.0 | 5 | 13.9 | 4.331 | 0.186 |
|  | Outcomes between treatment and control groups are the same | 17 | 6.5 | 2 | 5.6 |  |  |
|  | Both known and unknown prognostic factors are equally balanced between treatment and control patient groups | 172 | 65.6 | 27 | 75.0 |  |  |
|  | Patients receive the treatment or control that surgeons’ prefer the most | 5 | 1.9 | 2 | 5.6 |  |  |
|  | Never heard of it | 0 | 0.0 | 0 | 0.0 |  |  |
| In a randomized controlled study, how would you define double-blind? | Patients and outcome assessors are blind | 57 | 21.6 | 5 | 13.9 | 3.092 | 0.543 |
|  | Researcher and outcome assessors are blind | 48 | 18.2 | 8 | 22.2 |  |  |
|  | Patients and researchers are blind | 90 | 34.1 | 10 | 27.8 |  |  |
|  | Patients, researchers and outcome assessors are blind | 55 | 20.8 | 10 | 27.8 |  |  |
|  | I don’t have an idea | 14 | 5.3 | 3 | 8.3 |  |  |
| Do you believe a training for research methodology and evidence based medicine is necessary in neurosurgery? | Yes | 228 | 86.7 | 30 | 83.3 | 0.774 | 0.591 |
|  | Partially | 29 | 11.0 | 5 | 13.9 |  |  |
|  | No | 6 | 2.3 | 1 | 2.8 |  |  |
|  | Not Sure | 0 | 0.0 | 0 | 0.0 |  |  |
| Do you take into consideration the “level of evidence” or “grade of recommendation” provided by the study when you are reading a published scientific article? | Always | 94 | 35.9 | 12 | 33.3 | 0.143 | 0.963 |
|  | Frequently | 146 | 55.7 | 21 | 58.3 |  |  |
|  | Rarely | 22 | 8.4 | 3 | 8.3 |  |  |
|  | Never | 0 | 0.0 | 0 | 0.0 |  |  |
| Evidence-based medicine is used to.. | Make medical decisions correctly and scientifically for the patient’s benefit | 211 | 79.9 | 35 | 97.2 | 6.090 | 0.143 |
|  | Know the correct form of a manuscript | 20 | 7.6 | 0 | 0.0 |  |  |
|  | Learn what's new in the medical field | 18 | 6.8 | 0 | 0.0 |  |  |
|  | Disregard of evidence-based medicine in any individual study or current practice | 9 | 3.4 | 1 | 2.8 |  |  |
|  | I have not yet understood the usefulness of evidence-based medicine | 6 | 2.3 | 0 | 0.0 |  |  |

**Table 8. Comparison of Clinical Practice Setting in Responses in the Assessment of EBM**

|  | | **Clinical Practice Setting** | | | | | | | | **X^2^** | **p** |
| --- | --- | --- | --- | --- | --- | --- | --- | --- | --- | --- | --- |
|  |  | **Academic Practice** | | **Hospital Employment** | | **Private Practice** | | **Other** | |  |  |
|  |  | **n** | **%** | **n** | **%** | **n** | **%** | **n** | **%** |  |  |
| Are you familiar with the concept “evidence-based medicine” (EBM)? | I know and understand EBM but do not use it | 13 | 15.1 | 41 | 24.7 | 10 | 24.4 | 0 | 0.0 | 9.284 | 0.411 |
|  | I know and understand EBM and use it during my practice | 61 | 70.9 | 109 | 65.7 | 28 | 68.3 | 5 | 71.4 |  |  |
|  | I have heard of it but don’t understand what it is | 10 | 11.6 | 13 | 7.8 | 2 | 4.9 | 2 | 28.6 |  |  |
|  | I have never heard of it | 2 | 2.3 | 3 | 1.8 | 1 | 2.4 | 0 | 0.0 |  |  |
| Which of the following definitions do you think BEST defines “evidence-based medicine”? | Exclusive use of personal clinical experience of the practitioner in medical decision. | 5 | 5.8 | 10 | 6.0 | 3 | 7.3 | 1 | 14.3 | 3.449 | 0.944 |
|  | Exclusive use of medical research results in medical decision. | 22 | 25.6 | 30 | 18.1 | 7 | 17.1 | 1 | 14.3 |  |  |
|  | Combining personal clinical experience with the best evidence derived from medical scientific... | 57 | 66.3 | 121 | 72.9 | 30 | 73.2 | 5 | 71.4 |  |  |
|  | I do not know | 2 | 2.3 | 5 | 3.0 | 1 | 2.4 | 0 | 0.0 |  |  |
| Which of the following statements is FALSE concerning “VALIDITY”? | Validity includes two domains: internal and external validity. | 5 | 5.8 | 16 | 9.6 | 6 | 14.6 | 0 | 0.0 | 11.730 | 0.468 |
|  | Validity refers to how well the results represent true findings outside the study. | 22 | 25.6 | 49 | 29.5 | 9 | 22.0 | 0 | 0.0 |  |  |
|  | Internal and external validity can be both performed independent of each other. | 25 | 29.1 | 34 | 20.5 | 9 | 22.0 | 4 | 57.1 |  |  |
|  | A low external validity reduces the generalizability of the study findings. | 10 | 11.6 | 17 | 10.2 | 6 | 14.6 | 1 | 14.3 |  |  |
|  | I do not know | 24 | 27.9 | 50 | 30.1 | 11 | 26.8 | 2 | 28.6 |  |  |
| How would you define RANDOMIZATION? | Treatment and control patients are followed an equal amount of time and treated the same | 23 | 27.1 | 36 | 21.7 | 14 | 35.0 | 0 | 0.0 | 14.280 | 0.113 |
|  | Outcomes between treatment and control groups are the same | 2 | 2.4 | 13 | 7.8 | 3 | 7.5 | 1 | 14.3 |  |  |
|  | Both known and unknown prognostic factors are equally balanced between treatment and control patient groups | 57 | 67.1 | 114 | 68.7 | 23 | 57.5 | 5 | 71.4 |  |  |
|  | Patients receive the treatment or control that surgeons’ prefer the most | 3 | 3.5 | 3 | 1.8 | 0 | 0.0 | 1 | 14.3 |  |  |
| In a randomized controlled study, how would you define double-blind? | Patients and outcome assessors are blind | 20 | 23.3 | 31 | 18.7 | 11 | 26.8 | 0 | 0.0 | 8.440 | 0.750 |
|  | Researcher and outcome assessors are blind | 16 | 18.6 | 31 | 18.7 | 8 | 19.5 | 1 | 14.3 |  |  |
|  | Patients and researchers are blind | 24 | 27.9 | 62 | 37.3 | 12 | 29.3 | 2 | 28.6 |  |  |
|  | Patients, researchers and outcome assessors are blind | 22 | 25.6 | 32 | 19.3 | 8 | 19.5 | 3 | 42.9 |  |  |
|  | I don’t have an idea | 4 | 4.7 | 10 | 6.0 | 2 | 4.9 | 1 | 14.3 |  |  |
| Do you believe a training for research methodology and evidence based medicine is necessary in neurosurgery? | Yes | 80 | 93.0 | 143 | 86.1 | 31 | 75.6 | 4 | 66.7 | 11.405 | 0.077 |
|  | Partially | 4 | 4.7 | 19 | 11.4 | 9 | 22.0 | 2 | 33.3 |  |  |
|  | No | 2 | 2.3 | 4 | 2.4 | 1 | 2.4 | 0 | 0.0 |  |  |
| Do you take into consideration the “level of evidence” or “grade of recommendation” provided by the study when you are reading a published scientific article? | Always | 27 | 31.4 | 62 | 37.8 | 13 | 31.7 | 4 | 57.1 | 3.197 | 0.784 |
|  | Frequently | 51 | 59.3 | 88 | 53.7 | 25 | 61.0 | 3 | 42.9 |  |  |
|  | Rarely | 8 | 9.3 | 14 | 8.5 | 3 | 7.3 | 0 | 0.0 |  |  |
| Evidence-based medicine is used to.. | Make medical decisions correctly and scientifically for the patient’s benefit | 73 | 84.9 | 137 | 82.5 | 32 | 78.0 | 4 | 57.1 | 16.316 | 0.177 |
|  | Know the correct form of a manuscript | 6 | 7.0 | 9 | 5.4 | 5 | 12.2 | 0 | 0.0 |  |  |
|  | Learn what's new in the medical field | 5 | 5.8 | 9 | 5.4 | 3 | 7.3 | 1 | 14.3 |  |  |
|  | Disregard of evidence-based medicine in any individual study or current practice | 1 | 1.2 | 8 | 4.8 | 0 | 0.0 | 1 | 14.3 |  |  |
|  | I have not yet understood the usefulness of evidence-based medicine | 1 | 1.2 | 3 | 1.8 | 1 | 2.4 | 1 | 14.3 |  |  |

**Table 9. Comparison of Years of Medical Practice Setting in Responses in the Assessment of EBM**

|  | | **Years of Medical Practice (total)** | | | | | | | | | | **X^2^** | **p** |
| --- | --- | --- | --- | --- | --- | --- | --- | --- | --- | --- | --- | --- | --- |
|  |  | **Currently Training** | | **0-5** | | **6-10** | | **11-15** | | **15>** | |  |  |
|  |  | **n** | **%** | **n** | **%** | **n** | **%** | **n** | **%** | **n** | **%** |  |  |
| Are you familiar with the concept “evidence-based medicine” (EBM)? | I know and understand EBM but do not use it | 5 | 15.2 | 16 | 21.6 | 8 | 14.3 | 13 | 21.0 | 22 | 29.3 | 11.564 | 0.481 |
|  | I know and understand EBM and use it during my practice | 23 | 69.7 | 48 | 64.9 | 41 | 73.2 | 43 | 69.4 | 48 | 64.0 |  |  |
|  | I have heard of it but don’t understand what it is | 5 | 15.2 | 7 | 9.5 | 5 | 8.9 | 6 | 9.7 | 4 | 5.3 |  |  |
|  | I have never heard of it | 0 | 0.0 | 3 | 4.1 | 2 | 3.6 | 0 | 0.0 | 1 | 1.3 |  |  |
| Which of the following definitions do you think BEST defines “evidence-based medicine”? | Exclusive use of personal clinical experience of the practitioner in medical decision. | 5 | 15.2 | 5 | 6.8 | 0 | 0.0 | 4 | 6.5 | 5 | 6.7 | 17.538 | 0.130 |
|  | Exclusive use of medical research results in medical decision. | 4 | 12.1 | 10 | 13.5 | 13 | 23.2 | 11 | 17.7 | 22 | 29.3 |  |  |
|  | Combining personal clinical experience with the best evidence derived from medical scientific... | 23 | 69.7 | 56 | 75.7 | 41 | 73.2 | 45 | 72.6 | 48 | 64.0 |  |  |
|  | I do not know | 1 | 3.0 | 3 | 4.1 | 2 | 3.6 | 2 | 3.2 | 0 | 0.0 |  |  |
| Which of the following statements is FALSE concerning “VALIDITY”? | Validity includes two domains: internal and external validity. | 3 | 9.1 | 7 | 9.5 | 4 | 7.1 | 5 | 8.1 | 8 | 10.7 | 8.838 | 0.920 |
|  | Validity refers to how well the results represent true findings outside the study. | 7 | 21.2 | 14 | 18.9 | 19 | 33.9 | 18 | 29.0 | 22 | 29.3 |  |  |
|  | Internal and external validity can be both performed independent of each other. | 10 | 30.3 | 20 | 27.0 | 12 | 21.4 | 17 | 27.4 | 13 | 17.3 |  |  |
|  | A low external validity reduces the generalizability of the study findings. | 5 | 15.2 | 8 | 10.8 | 5 | 8.9 | 7 | 11.3 | 9 | 12.0 |  |  |
|  | I do not know | 8 | 24.2 | 25 | 33.8 | 16 | 28.6 | 15 | 24.2 | 23 | 30.7 |  |  |
| How would you define RANDOMIZATION? | Treatment and control patients are followed an equal amount of time and treated the same | 8 | 24.2 | 13 | 17.8 | 16 | 28.6 | 13 | 21.0 | 23 | 31.1 | 13.110 | 0.361 |
|  | Outcomes between treatment and control groups are the same | 1 | 3.0 | 5 | 6.8 | 3 | 5.4 | 7 | 11.3 | 3 | 4.1 |  |  |
|  | Both known and unknown prognostic factors are equally balanced between treatment and control patient groups | 24 | 72.7 | 51 | 69.9 | 37 | 66.1 | 40 | 64.5 | 47 | 63.5 |  |  |
|  | Patients receive the treatment or control that surgeons’ prefer the most | 0 | 0.0 | 4 | 5.5 | 0 | 0.0 | 2 | 3.2 | 1 | 1.4 |  |  |
| In a randomized controlled study, how would you define double-blind? | Patients and outcome assessors are blind | 4 | 12.1 | 20 | 27.0 | 10 | 17.9 | 13 | 21.0 | 15 | 20.0 | 13.058 | 0.668 |
|  | Researcher and outcome assessors are blind | 5 | 15.2 | 11 | 14.9 | 11 | 19.6 | 15 | 24.2 | 14 | 18.7 |  |  |
|  | Patients and researchers are blind | 12 | 36.4 | 25 | 33.8 | 20 | 35.7 | 17 | 27.4 | 26 | 34.7 |  |  |
|  | Patients, researchers and outcome assessors are blind | 11 | 33.3 | 11 | 14.9 | 13 | 23.2 | 15 | 24.2 | 15 | 20.0 |  |  |
|  | I don’t have an idea | 1 | 3.0 | 7 | 9.5 | 2 | 3.6 | 2 | 3.2 | 5 | 6.7 |  |  |
| Do you believe a training for research methodology and evidence based medicine is necessary in neurosurgery? | Yes | 32 | 97.0 | 66 | 89.2 | 51 | 91.1 | 50 | 82.0 | 59 | 78.7 | 12.160 | 0.144 |
|  | Partially | 0 | 0.0 | 6 | 8.1 | 5 | 8.9 | 10 | 16.4 | 13 | 17.3 |  |  |
|  | No | 1 | 3.0 | 2 | 2.7 | 0 | 0.0 | 1 | 1.6 | 3 | 4.0 |  |  |
| Do you take into consideration the “level of evidence” or “grade of recommendation” provided by the study when you are reading a published scientific article? | Always | 15 | 45.5 | 26 | 35.6 | 20 | 36.4 | 22 | 35.5 | 23 | 30.7 | 7.427 | 0.491 |
|  | Frequently | 18 | 54.5 | 43 | 58.9 | 30 | 54.5 | 34 | 54.8 | 42 | 56.0 |  |  |
|  | Rarely | 0 | 0.0 | 4 | 5.5 | 5 | 9.1 | 6 | 9.7 | 10 | 13.3 |  |  |
| Evidence-based medicine is used to.. | Make medical decisions correctly and scientifically for the patient’s benefit | 27 | 81.8 | 54 | 73.0 | 48 | 85.7 | 54 | 87.1 | 63 | 84.0 | x | x |
|  | Know the correct form of a manuscript | 2 | 6.1 | 7 | 9.5 | 3 | 5.4 | 4 | 6.5 | 4 | 5.3 |  |  |
|  | Learn what's new in the medical field | 2 | 6.1 | 5 | 6.8 | 3 | 5.4 | 2 | 3.2 | 6 | 8.0 |  |  |
|  | Disregard of evidence-based medicine in any individual study or current practice | 2 | 6.1 | 4 | 5.4 | 2 | 3.6 | 1 | 1.6 | 1 | 1.3 |  |  |
|  | I have not yet understood the usefulness of evidence-based medicine | 0 | 0.0 | 4 | 5.4 | 0 | 0.0 | 1 | 1.6 | 1 | 1.3 |  |  |

**Table 10. Comparison of Training in Responses in the Assessment of EBM**

|  | | **Have you received additional training/education/fellowship/subspecialty for Spine Surgery?** | | | | **X^2^** | **p** |
| --- | --- | --- | --- | --- | --- | --- | --- |
|  |  | **Yes** | | **No** | |  |  |
|  |  | **n** | **%** | **n** | **%** |  |  |
| Are you familiar with the concept “evidence-based medicine” (EBM)? | I know and understand EBM but do not use it | 24 | 20.0 | 40 | 22.2 | 2.016 | 0.579 |
|  | I know and understand EBM and use it during my practice | 82 | 68.3 | 121 | 67.2 |  |  |
|  | I have heard of it but don’t understand what it is | 10 | 8.3 | 17 | 9.4 |  |  |
|  | I have never heard of it | 4 | 3.3 | 2 | 1.1 |  |  |
| Which of the following definitions do you think BEST defines “evidence-based medicine”? | Exclusive use of personal clinical experience of the practitioner in medical decision. | 7 | 5.8 | 12 | 6.7 | 0.168 | 0.994 |
|  | Exclusive use of medical research results in medical decision. | 24 | 20.0 | 36 | 20.0 |  |  |
|  | Combining personal clinical experience with the best evidence derived from medical scientific... | 86 | 71.7 | 127 | 70.6 |  |  |
|  | I do not know | 3 | 2.5 | 5 | 2.8 |  |  |
| Which of the following statements is FALSE concerning “VALIDITY”? | Validity includes two domains: internal and external validity. | 8 | 6.7 | 19 | 10.6 | 1.713 | 0.796 |
|  | Validity refers to how well the results represent true findings outside the study. | 32 | 26.7 | 48 | 26.7 |  |  |
|  | Internal and external validity can be both performed independent of each other. | 28 | 23.3 | 44 | 24.4 |  |  |
|  | A low external validity reduces the generalizability of the study findings. | 15 | 12.5 | 19 | 10.6 |  |  |
|  | I do not know | 37 | 30.8 | 50 | 27.8 |  |  |
| How would you define RANDOMIZATION? | Treatment and control patients are followed an equal amount of time and treated the same | 27 | 22.7 | 46 | 25.7 | 1.680 | 0.663 |
|  | Outcomes between treatment and control groups are the same | 9 | 7.6 | 10 | 5.6 |  |  |
|  | Both known and unknown prognostic factors are equally balanced between treatment and control patient groups | 79 | 66.4 | 120 | 67.0 |  |  |
|  | Patients receive the treatment or control that surgeons’ prefer the most | 4 | 3.4 | 3 | 1.7 |  |  |
| In a randomized controlled study, how would you define double-blind? | Patients and outcome assessors are blind | 22 | 18.3 | 40 | 22.2 | 2.732 | 0.608 |
|  | Researcher and outcome assessors are blind | 20 | 16.7 | 36 | 20.0 |  |  |
|  | Patients and researchers are blind | 40 | 33.3 | 60 | 33.3 |  |  |
|  | Patients, researchers and outcome assessors are blind | 29 | 24.2 | 36 | 20.0 |  |  |
|  | I don’t have an idea | 9 | 7.5 | 8 | 4.4 |  |  |
| Do you believe a training for research methodology and evidence based medicine is necessary in neurosurgery? | Yes | 98 | 82.4 | 160 | 88.9 | 2.907 | 0.244 |
|  | Partially | 18 | 15.1 | 16 | 8.9 |  |  |
|  | No | 3 | 2.5 | 4 | 2.2 |  |  |
| Do you take into consideration the “level of evidence” or “grade of recommendation” provided by the study when you are reading a published scientific article? | Always | 41 | 34.7 | 65 | 36.1 | 0.848 | 0.664 |
|  | Frequently | 65 | 55.1 | 102 | 56.7 |  |  |
|  | Rarely | 12 | 10.2 | 13 | 7.2 |  |  |
| Evidence-based medicine is used to.. | Make medical decisions correctly and scientifically for the patient’s benefit | 102 | 85.0 | 144 | 80.0 | 3.118 | 0.534 |
|  | Know the correct form of a manuscript | 7 | 5.8 | 13 | 7.2 |  |  |
|  | Learn what's new in the medical field | 4 | 3.3 | 14 | 7.8 |  |  |
|  | Disregard of evidence-based medicine in any individual study or current practice | 4 | 3.3 | 6 | 3.3 |  |  |
|  | I have not yet understood the usefulness of evidence-based medicine | 3 | 2.5 | 3 | 1.7 |  |  |

**Table 11. Comparison of Responses Amongst Genders to Various Clinical Scenarios**

|  | | **Gender** | | | | **X^2^** | **p** |
| --- | --- | --- | --- | --- | --- | --- | --- |
|  |  | **Male** | | **Female** | |  |  |
|  |  | **n** | **%** | **n** | **%** |  |  |
| Do you adhere to any guidelines or scoring systems when making decisions in spinal pathologies? | Always | 117 | 45.0 | 15 | 42.9 | 2.230 | 0.324 |
|  | Frequently | 134 | 51.5 | 17 | 48.6 |  |  |
|  | Rarely | 9 | 3.5 | 3 | 8.6 |  |  |
| In patients where no guideline or scoring system is used, what is your decision mostly based on? | Personal experience | 132 | 50.0 | 16 | 44.4 | 0.391 | 0.596 |
|  | Face-to-face consultation with neurosurgeons | 114 | 43.2 | 19 | 52.8 | 1.182 | 0.289 |
|  | Social media collaboration with neurosurgeons | 39 | 14.8 | 2 | 5.6 | 2.281 | 0.194 |
|  | Literature search | 144 | 54.5 | 21 | 58.3 | 0.184 | 0.723 |
|  | Reference text-books | 83 | 31.4 | 15 | 41.7 | 1.077 | 0.299 |
| In a patient applying to your clinic with severe low back pain that has affected their quality of life with no red-flag signs (radiculopathy, bladder problems, deficits etc.) detected during examination, what is your FIRST step? | Obtain X-ray/CT | 57 | 21.6 | 12 | 33.3 | 3.118 | 0.538 |
|  | Obtain MRI | 97 | 36.7 | 12 | 33.3 |  |  |
|  | Prescribe medication | 84 | 31.8 | 9 | 25.0 |  |  |
|  | Suggest bed-rest | 15 | 5.7 | 1 | 2.8 |  |  |
|  | None | 11 | 4.2 | 2 | 5.6 |  |  |
| Do you believe the choice of surgical approach (anterior, posterior, or combined anterior posterior) improve clinical outcomes in patients with thoracic and lumbar fractures? | Always | 104 | 39.4 | 12 | 33.3 | 1.069 | 0.805 |
|  | Sometimes | 142 | 53.8 | 21 | 58.3 |  |  |
|  | Never | 8 | 3.0 | 1 | 2.8 |  |  |
|  | Not Sure | 10 | 3.8 | 2 | 5.6 |  |  |
| Do you believe the addition of arthrodesis to instrumented fixation improve outcomes in patients with thoracic and lumbar burst fractures? | Always | 83 | 31.4 | 8 | 22.2 | 1.671 | 0.629 |
|  | Sometimes | 151 | 57.2 | 25 | 69.4 |  |  |
|  | Never | 10 | 3.8 | 1 | 2.8 |  |  |
|  | Not Sure | 20 | 7.6 | 2 | 5.6 |  |  |
| Do you treat low BMD (Bone Mineral Density T <-2.5) before spine surgery? | Always | 67 | 25.4 | 16 | 44.4 | 6.367 | 0.082 |
|  | Sometimes | 145 | 54.9 | 13 | 36.1 |  |  |
|  | Never | 31 | 11.7 | 5 | 13.9 |  |  |
|  | Not Sure | 21 | 8.0 | 2 | 5.6 |  |  |
| Do you prescribe high dose methylprednisolone to patients presenting with acute spinal cord injury with neurological findings? | Always | 92 | 35.0 | 8 | 22.9 | 4.409 | 0.193 |
|  | Sometimes | 87 | 33.1 | 18 | 51.4 |  |  |
|  | Never | 75 | 28.5 | 8 | 22.9 |  |  |
|  | Not Sure | 9 | 3.4 | 1 | 2.9 |  |  |
| Do you perform fusion in patients treated for lumbar stenosis with or without spondylolisthesis after decompression? | Always | 48 | 18.2 | 6 | 16.7 | 4.014 | 0.227 |
|  | Sometimes | 183 | 69.3 | 22 | 61.1 |  |  |
|  | Never | 26 | 9.8 | 5 | 13.9 |  |  |
|  | Not Sure | 7 | 2.7 | 3 | 8.3 |  |  |
| Which of the following statements about the surgical treatment of cervical radiculopathy do you agree with the most? | When correcting cervical sagittal alignment, ACDF alone is sufficient. | 95 | 36.0 | 5 | 13.9 | 10.525 | **0.013** |
|  | Anterior surgery results in better outcomes than posterior surgery | 78 | 29.5 | 11 | 30.6 |  |  |
|  | TDA results in better outcomes than ACDF when treating single level soft disc herniations. | 36 | 13.6 | 5 | 13.9 |  |  |
|  | The Neck Disability Index, SF-36, SF-12 and VAS are recommended outcome measures for assessing ... | 55 | 20.8 | 15 | 41.7 |  |  |
| What is your recommendation to a patient with unilateral extruded disc herniation with no leg pain at the time of your examination? | Microdiscectomy | 43 | 16.3 | 4 | 11.1 | 0.623 | 0.917 |
|  | Physical therapy | 81 | 30.7 | 12 | 33.3 |  |  |
|  | Medical therapy | 98 | 37.1 | 14 | 38.9 |  |  |
|  | No treatment | 42 | 15.9 | 6 | 16.7 |  |  |

**Table 12. Comparison of Region of Practice in Assesment of EBM**

|  | | **Region of practice** | | | | | | | | | | **X^2^** | **p** |
| --- | --- | --- | --- | --- | --- | --- | --- | --- | --- | --- | --- | --- | --- |
|  |  | **North America** | | **South America** | | **Europe** | | **Asia** | | **Africa** | |  |  |
|  |  | **n** | **%** | **n** | **%** | **n** | **%** | **n** | **%** | **n** | **%** |  |  |
| Are you familiar with the concept “evidence-based medicine” (EBM)? | I know and understand EBM but do not use it | 1 | 5.3 | 9 | 27.3 | 14 | 23.3 | 30 | 20.0 | 10 | 27.0 | 23.100 | **0.027** |
|  | I know and understand EBM and use it during my practice | 18 | 94.7 | 23 | 69.7 | 38 | 63.3 | 103 | 68.7 | 20 | 54.1 |  |  |
|  | I have heard of it but don’t understand what it is | 0 | 0.0 | 1 | 3.0 | 4 | 6.7 | 15 | 10.0 | 7 | 18.9 |  |  |
|  | I have never heard of it | 0 | 0.0 | 0 | 0.0 | 4 | 6.7 | 2 | 1.3 | 0 | 0.0 |  |  |
| Which of the following definitions do you think BEST defines “evidence-based medicine”? | Exclusive use of personal clinical experience of the practitioner in medical decision. | 0 | 0.0 | 4 | 12.1 | 2 | 3.3 | 10 | 6.7 | 3 | 8.1 | 15.087 | 0.237 |
|  | Exclusive use of medical research results in medical decision. | 3 | 15.8 | 8 | 24.2 | 15 | 25.0 | 24 | 16.0 | 10 | 27.0 |  |  |
|  | Combining personal clinical experience with the best evidence derived from medical scientific... | 16 | 84.2 | 21 | 63.6 | 42 | 70.0 | 112 | 74.7 | 21 | 56.8 |  |  |
|  | I do not know | 0 | 0.0 | 0 | 0.0 | 1 | 1.7 | 4 | 2.7 | 3 | 8.1 |  |  |
| Which of the following statements is FALSE concerning “VALIDITY”? | Validity includes two domains: internal and external validity. | 2 | 10.5 | 4 | 12.1 | 6 | 10.0 | 12 | 8.0 | 3 | 8.1 | 7.575 | 0.961 |
|  | Validity refers to how well the results represent true findings outside the study. | 6 | 31.6 | 9 | 27.3 | 19 | 31.7 | 36 | 24.0 | 10 | 27.0 |  |  |
|  | Internal and external validity can be both performed independent of each other. | 4 | 21.1 | 8 | 24.2 | 14 | 23.3 | 38 | 25.3 | 8 | 21.6 |  |  |
|  | A low external validity reduces the generalizability of the study findings. | 2 | 10.5 | 5 | 15.2 | 2 | 3.3 | 20 | 13.3 | 4 | 10.8 |  |  |
|  | I do not know | 5 | 26.3 | 7 | 21.2 | 19 | 31.7 | 44 | 29.3 | 12 | 32.4 |  |  |
| How would you define RANDOMIZATION? | Treatment and control patients are followed an equal amount of time and treated the same | 5 | 26.3 | 8 | 24.2 | 11 | 18.6 | 39 | 26.2 | 10 | 27.0 | 11.194 | 0.512 |
|  | Outcomes between treatment and control groups are the same | 2 | 10.5 | 4 | 12.1 | 1 | 1.7 | 8 | 5.4 | 4 | 10.8 |  |  |
|  | Both known and unknown prognostic factors are equally balanced between treatment and control patient groups | 12 | 63.2 | 20 | 60.6 | 45 | 76.3 | 100 | 67.1 | 21 | 56.8 |  |  |
|  | Patients receive the treatment or control that surgeons’ prefer the most | 0 | 0.0 | 1 | 3.0 | 2 | 3.4 | 2 | 1.3 | 2 | 5.4 |  |  |
| In a randomized controlled study, how would you define double-blind? | Patients and outcome assessors are blind | 4 | 21.1 | 8 | 24.2 | 12 | 20.0 | 30 | 20.0 | 8 | 21.6 | 17.701 | 0.342 |
|  | Researcher and outcome assessors are blind | 4 | 21.1 | 9 | 27.3 | 7 | 11.7 | 28 | 18.7 | 8 | 21.6 |  |  |
|  | Patients and researchers are blind | 10 | 52.6 | 10 | 30.3 | 24 | 40.0 | 44 | 29.3 | 11 | 29.7 |  |  |
|  | Patients, researchers and outcome assessors are blind | 1 | 5.3 | 5 | 15.2 | 16 | 26.7 | 37 | 24.7 | 6 | 16.2 |  |  |
|  | I don’t have an idea | 0 | 0.0 | 1 | 3.0 | 1 | 1.7 | 11 | 7.3 | 4 | 10.8 |  |  |
| Do you believe a training for research methodology and evidence based medicine is necessary in neurosurgery? | Yes | 16 | 84.2 | 26 | 81.3 | 53 | 88.3 | 129 | 86.0 | 33 | 89.2 | 10.221 | 0.250 |
|  | Partially | 1 | 5.3 | 6 | 18.8 | 5 | 8.3 | 18 | 12.0 | 4 | 10.8 |  |  |
|  | No | 2 | 10.5 | 0 | 0.0 | 2 | 3.3 | 3 | 2.0 | 0 | 0.0 |  |  |
| Do you take into consideration the “level of evidence” or “grade of recommendation” provided by the study when you are reading a published scientific article? | Always | 6 | 31.6 | 8 | 24.2 | 16 | 27.1 | 67 | 45.0 | 9 | 24.3 | 13.129 | 0.107 |
|  | Frequently | 12 | 63.2 | 22 | 66.7 | 37 | 62.7 | 70 | 47.0 | 26 | 70.3 |  |  |
|  | Rarely | 1 | 5.3 | 3 | 9.1 | 6 | 10.2 | 12 | 8.1 | 2 | 5.4 |  |  |
| Evidence-based medicine is used to.. | Make medical decisions correctly and scientifically for the patient’s benefit | 17 | 89.5 | 27 | 81.8 | 48 | 80.0 | 122 | 81.3 | 31 | 83.8 | 8.159 | 0.944 |
|  | Know the correct form of a manuscript | 1 | 5.3 | 3 | 9.1 | 4 | 6.7 | 11 | 7.3 | 1 | 2.7 |  |  |
|  | Learn what's new in the medical field | 1 | 5.3 | 1 | 3.0 | 4 | 6.7 | 8 | 5.3 | 4 | 10.8 |  |  |
|  | Disregard of evidence-based medicine in any individual study or current practice | 0 | 0.0 | 2 | 6.1 | 3 | 5.0 | 5 | 3.3 | 0 | 0.0 |  |  |
|  | I have not yet understood the usefulness of evidence-based medicine | 0 | 0.0 | 0 | 0.0 | 1 | 1.7 | 4 | 2.7 | 1 | 2.7 |  |  |

**Table 13. Comparison of Responses Amongst Age Groups to Various Clinical Scenarios**

|  | | **Age (years)** | | | | | | | | **X^2^** | **p** |
| --- | --- | --- | --- | --- | --- | --- | --- | --- | --- | --- | --- |
|  |  | **≤30** | | **31-40** | | **41-50** | | **50>** | |  |  |
|  |  | **n** | **%** | **n** | **%** | **n** | **%** | **n** | **%** |  |  |
| Do you adhere to any guidelines or scoring systems when making decisions in spinal pathologies? | Always | 20 | 52.6 | 67 | 48.9 | 33 | 40.7 | 12 | 30.8 | 7.026 | 0.318 |
|  | Frequently | 17 | 44.7 | 63 | 46.0 | 46 | 56.8 | 25 | 64.1 |  |  |
|  | Rarely | 1 | 2.6 | 7 | 5.1 | 2 | 2.5 | 2 | 5.1 |  |  |
| In patients where no guideline or scoring system is used, what is your decision mostly based on? | Personal experience | 24 | 61.5 | 56 | 40.6 | 47 | 57.3 | 21 | 51.2 | 8.704 | **0.033** |
|  | Face-to-face consultation with neurosurgeons | 26 | 66.7 | 61 | 44.2 | 30 | 36.6 | 16 | 39.0 | 10.346 | **0.016** |
|  | Social media collaboration with neurosurgeons | 6 | 15.4 | 18 | 13.0 | 12 | 14.6 | 5 | 12.2 | 0.283 | 0.963 |
|  | Literature search | 21 | 53.8 | 81 | 58.7 | 42 | 51.2 | 21 | 51.2 | 1.493 | 0.684 |
|  | Reference text-books | 13 | 33.3 | 50 | 36.2 | 24 | 29.3 | 11 | 26.8 | 1.871 | 0.600 |
| In a patient applying to your clinic with severe low back pain that has affected their quality of life with no red-flag signs (radiculopathy, bladder problems, deficits etc.) detected during examination, what is your FIRST step? | Obtain X-ray/CT | 11 | 28.2 | 35 | 25.4 | 17 | 20.7 | 6 | 14.6 | 12.311 | 0.421 |
|  | Obtain MRI | 14 | 35.9 | 43 | 31.2 | 33 | 40.2 | 19 | 46.3 |  |  |
|  | Prescribe medication | 10 | 25.6 | 49 | 35.5 | 24 | 29.3 | 10 | 24.4 |  |  |
|  | Suggest bed-rest | 1 | 2.6 | 8 | 5.8 | 3 | 3.7 | 4 | 9.8 |  |  |
|  | None | 3 | 7.7 | 3 | 2.2 | 5 | 6.1 | 2 | 4.9 |  |  |
| Do you believe the choice of surgical approach (anterior, posterior, or combined anterior posterior) improve clinical outcomes in patients with thoracic and lumbar fractures? | Always | 15 | 38.5 | 54 | 39.1 | 30 | 36.6 | 17 | 41.5 | 8.451 | 0.489 |
|  | Sometimes | 22 | 56.4 | 70 | 50.7 | 49 | 59.8 | 22 | 53.7 |  |  |
|  | Never | 0 | 0.0 | 5 | 3.6 | 2 | 2.4 | 2 | 4.9 |  |  |
|  | Not Sure | 2 | 5.1 | 9 | 6.5 | 1 | 1.2 | 0 | 0.0 |  |  |
| Do you believe the addition of arthrodesis to instrumented fixation improve outcomes in patients with thoracic and lumbar burst fractures? | Always | 5 | 12.8 | 41 | 29.7 | 30 | 36.6 | 15 | 36.6 | 19.208 | **0.023** |
|  | Sometimes | 29 | 74.4 | 80 | 58.0 | 49 | 59.8 | 18 | 43.9 |  |  |
|  | Never | 2 | 5.1 | 6 | 4.3 | 2 | 2.4 | 1 | 2.4 |  |  |
|  | Not Sure | 3 | 7.7 | 11 | 8.0 | 1 | 1.2 | 7 | 17.1 |  |  |
| Do you treat low BMD (Bone Mineral Density T <-2.5) before spine surgery? | Always | 18 | 46.2 | 33 | 23.9 | 25 | 30.5 | 7 | 17.1 | 18.407 | **0.031** |
|  | Sometimes | 12 | 30.8 | 80 | 58.0 | 39 | 47.6 | 27 | 65.9 |  |  |
|  | Never | 3 | 7.7 | 17 | 12.3 | 12 | 14.6 | 4 | 9.8 |  |  |
|  | Not Sure | 6 | 15.4 | 8 | 5.8 | 6 | 7.3 | 3 | 7.3 |  |  |
| Do you prescribe high dose methylprednisolone to patients presenting with acute spinal cord injury with neurological findings? | Always | 12 | 30.8 | 52 | 38.2 | 23 | 28.0 | 13 | 31.7 | 10.418 | 0.318 |
|  | Sometimes | 19 | 48.7 | 47 | 34.6 | 26 | 31.7 | 13 | 31.7 |  |  |
|  | Never | 6 | 15.4 | 33 | 24.3 | 30 | 36.6 | 14 | 34.1 |  |  |
|  | Not Sure | 2 | 5.1 | 4 | 2.9 | 3 | 3.7 | 1 | 2.4 |  |  |
| Do you perform fusion in patients treated for lumbar stenosis with or without spondylolisthesis after decompression? | Always | 6 | 15.4 | 30 | 21.7 | 12 | 14.6 | 6 | 14.6 | 19.786 | **0.019** |
|  | Sometimes | 25 | 64.1 | 93 | 67.4 | 57 | 69.5 | 30 | 73.2 |  |  |
|  | Never | 3 | 7.7 | 12 | 8.7 | 13 | 15.9 | 3 | 7.3 |  |  |
|  | Not Sure | 5 | 12.8 | 3 | 2.2 | 0 | 0.0 | 2 | 4.9 |  |  |
| Which of the following statements about the surgical treatment of cervical radiculopathy do you agree with the most? | When correcting cervical sagittal alignment, ACDF alone is sufficient. | 11 | 28.2 | 45 | 32.6 | 28 | 34.1 | 16 | 39.0 | 6.408 | 0.698 |
|  | Anterior surgery results in better outcomes than posterior surgery | 11 | 28.2 | 46 | 33.3 | 18 | 22.0 | 14 | 34.1 |  |  |
|  | TDA results in better outcomes than ACDF when treating single level soft disc herniations. | 6 | 15.4 | 17 | 12.3 | 14 | 17.1 | 4 | 9.8 |  |  |
|  | The Neck Disability Index, SF-36, SF-12 and VAS are recommended outcome measures for assessing ... | 11 | 28.2 | 30 | 21.7 | 22 | 26.8 | 7 | 17.1 |  |  |
| What is your recommendation to a patient with unilateral extruded disc herniation with no leg pain at the time of your examination? | Microdiscectomy | 6 | 15.4 | 20 | 14.5 | 12 | 14.6 | 9 | 22.0 | 4.896 | 0.843 |
|  | Physical therapy | 14 | 35.9 | 41 | 29.7 | 27 | 32.9 | 11 | 26.8 |  |  |
|  | Medical therapy | 16 | 41.0 | 52 | 37.7 | 31 | 37.8 | 13 | 31.7 |  |  |
|  | No treatment | 3 | 7.7 | 25 | 18.1 | 12 | 14.6 | 8 | 19.5 |  |  |

**Table 14. Comparison of Status in Various Clinical Scenarios**

|  | | **Status** | | | | | | | | **X^2^** | **p** |
| --- | --- | --- | --- | --- | --- | --- | --- | --- | --- | --- | --- |
|  |  | **Resident** | | **Consultant** | | **Academic** | | **Other** | |  |  |
|  |  | **n** | **%** | **n** | **%** | **n** | **%** | **n** | **%** |  |  |
| Do you adhere to any guidelines or scoring systems when making decisions in spinal pathologies? | Always | 35 | 42.7 | 68 | 47.6 | 22 | 42.3 | 7 | 38.9 | 10.523 | 0.104 |
|  | Frequently | 44 | 53.7 | 72 | 50.3 | 24 | 46.2 | 11 | 61.1 |  |  |
|  | Rarely | 3 | 3.7 | 3 | 2.1 | 6 | 11.5 | 0 | 0.0 |  |  |
| In patients where no guideline or scoring system is used, what is your decision mostly based on? | Personal experience | 43 | 51.2 | 75 | 51.7 | 19 | 36.5 | 11 | 57.9 | 4.41 | 0.220 |
|  | Face-to-face consultation with neurosurgeons | 44 | 52.4 | 55 | 37.9 | 22 | 42.3 | 12 | 63.2 | 7.427 | 0.059 |
|  | Social media collaboration with neurosurgeons | 8 | 9.5 | 20 | 13.8 | 10 | 19.2 | 3 | 15.8 | 2.661 | 0.447 |
|  | Literature search | 47 | 56.0 | 72 | 49.7 | 33 | 63.5 | 13 | 68.4 | 4.591 | 0.204 |
|  | Reference text-books | 29 | 34.5 | 43 | 29.7 | 19 | 36.5 | 7 | 36.8 | 1.235 | 0.745 |
| In a patient applying to your clinic with severe low back pain that has affected their quality of life with no red-flag signs (radiculopathy, bladder problems, deficits etc.) detected during examination, what is your FIRST step? | Obtain X-ray/CT | 25 | 29.8 | 34 | 23.4 | 6 | 11.5 | 4 | 21.1 | 12.837 | 0.381 |
|  | Obtain MRI | 21 | 25.0 | 55 | 37.9 | 25 | 48.1 | 8 | 42.1 |  |  |
|  | Prescribe medication | 28 | 33.3 | 42 | 29.0 | 18 | 34.6 | 5 | 26.3 |  |  |
|  | Suggest bed-rest | 5 | 6.0 | 9 | 6.2 | 1 | 1.9 | 1 | 5.3 |  |  |
|  | None | 5 | 6.0 | 5 | 3.4 | 2 | 3.8 | 1 | 5.3 |  |  |
| Do you believe the choice of surgical approach (anterior, posterior, or combined anterior posterior) improve clinical outcomes in patients with thoracic and lumbar fractures? | Always | 35 | 41.7 | 53 | 36.6 | 20 | 38.5 | 8 | 42.1 | 5.445 | 0.794 |
|  | Sometimes | 43 | 51.2 | 82 | 56.6 | 28 | 53.8 | 10 | 52.6 |  |  |
|  | Never | 1 | 1.2 | 4 | 2.8 | 3 | 5.8 | 1 | 5.3 |  |  |
|  | Not Sure | 5 | 6.0 | 6 | 4.1 | 1 | 1.9 | 0 | 0.0 |  |  |
| Do you believe the addition of arthrodesis to instrumented fixation improve outcomes in patients with thoracic and lumbar burst fractures? | Always | 19 | 22.6 | 44 | 30.3 | 23 | 44.2 | 5 | 26.3 | 13.183 | 0.154 |
|  | Sometimes | 57 | 67.9 | 81 | 55.9 | 26 | 50.0 | 12 | 63.2 |  |  |
|  | Never | 2 | 2.4 | 9 | 6.2 | 0 | 0.0 | 0 | 0.0 |  |  |
|  | Not Sure | 6 | 7.1 | 11 | 7.6 | 3 | 5.8 | 2 | 10.5 |  |  |
| Do you treat low BMD (Bone Mineral Density T <-2.5) before spine surgery? | Always | 24 | 28.6 | 40 | 27.6 | 15 | 28.8 | 4 | 21.1 | 7.392 | 0.596 |
|  | Sometimes | 39 | 46.4 | 82 | 56.6 | 27 | 51.9 | 10 | 52.6 |  |  |
|  | Never | 12 | 14.3 | 17 | 11.7 | 4 | 7.7 | 3 | 15.8 |  |  |
|  | Not Sure | 9 | 10.7 | 6 | 4.1 | 6 | 11.5 | 2 | 10.5 |  |  |
| Do you prescribe high dose methylprednisolone to patients presenting with acute spinal cord injury with neurological findings? | Always | 29 | 34.5 | 48 | 33.3 | 17 | 33.3 | 6 | 31.6 | 5.982 | 0.742 |
|  | Sometimes | 32 | 38.1 | 48 | 33.3 | 19 | 37.3 | 6 | 31.6 |  |  |
|  | Never | 19 | 22.6 | 46 | 31.9 | 12 | 23.5 | 6 | 31.6 |  |  |
|  | Not Sure | 4 | 4.8 | 2 | 1.4 | 3 | 5.9 | 1 | 5.3 |  |  |
| Do you perform fusion in patients treated for lumbar stenosis with or without spondylolisthesis after decompression? | Always | 18 | 21.4 | 23 | 15.9 | 10 | 19.2 | 3 | 15.8 | 17.240 | **0.045** |
|  | Sometimes | 52 | 61.9 | 101 | 69.7 | 38 | 73.1 | 14 | 73.7 |  |  |
|  | Never | 9 | 10.7 | 20 | 13.8 | 0 | 0.0 | 2 | 10.5 |  |  |
|  | Not Sure | 5 | 6.0 | 1 | 0.7 | 4 | 7.7 | 0 | 0.0 |  |  |
| Which of the following statements about the surgical treatment of cervical radiculopathy do you agree with the most? | When correcting cervical sagittal alignment, ACDF alone is sufficient. | 27 | 32.1 | 46 | 31.7 | 24 | 46.2 | 3 | 15.8 | 9.825 | 0.365 |
|  | Anterior surgery results in better outcomes than posterior surgery | 25 | 29.8 | 46 | 31.7 | 9 | 17.3 | 9 | 47.4 |  |  |
|  | TDA results in better outcomes than ACDF when treating single level soft disc herniations. | 13 | 15.5 | 19 | 13.1 | 7 | 13.5 | 2 | 10.5 |  |  |
|  | The Neck Disability Index, SF-36, SF-12 and VAS are recommended outcome measures for assessing ... | 19 | 22.6 | 34 | 23.4 | 12 | 23.1 | 5 | 26.3 |  |  |
| What is your recommendation to a patient with unilateral extruded disc herniation with no leg pain at the time of your examination? | Microdiscectomy | 13 | 15.5 | 18 | 12.4 | 11 | 21.2 | 5 | 26.3 | 10.643 | 0.301 |
|  | Physical therapy | 33 | 39.3 | 42 | 29.0 | 15 | 28.8 | 3 | 15.8 |  |  |
|  | Medical therapy | 24 | 28.6 | 63 | 43.4 | 17 | 32.7 | 8 | 42.1 |  |  |
|  | No treatment | 14 | 16.7 | 22 | 15.2 | 9 | 17.3 | 3 | 15.8 |  |  |

**Table 15. Comparison of Clinical Practice Setting in Various Clinical Scenarios**

|  | | **Clinical Practice Setting** | | | | | | | | **X^2^** | **p** |
| --- | --- | --- | --- | --- | --- | --- | --- | --- | --- | --- | --- |
|  |  | **Academic Practice** | | **Hospital Employment** | | **Private Practice** | | **Other** | |  |  |
|  |  | **n** | **%** | **n** | **%** | **n** | **%** | **n** | **%** |  |  |
| Do you adhere to any guidelines or scoring systems when making decisions in spinal pathologies? | Always | 35 | 42.2 | 76 | 45.8 | 17 | 43.6 | 4 | 57.1 | 2.089 | 0.911 |
|  | Frequently | 46 | 55.4 | 82 | 49.4 | 20 | 51.3 | 3 | 42.9 |  |  |
|  | Rarely | 2 | 2.4 | 8 | 4.8 | 2 | 5.1 | 0 | 0.0 |  |  |
| In patients where no guideline or scoring system is used, what is your decision mostly based on? | Personal experience | 39 | 45.3 | 84 | 50.6 | 24 | 58.5 | 1 | 14.3 | 5.482 | 0.140 |
|  | Face-to-face consultation with neurosurgeons | 32 | 37.2 | 87 | 52.4 | 12 | 29.3 | 2 | 28.6 | 10.631 | **0.014** |
|  | Social media collaboration with neurosurgeons | 8 | 9.3 | 22 | 13.3 | 8 | 19.5 | 3 | 42.9 | 7.655 | 0.054 |
|  | Literature search | 58 | 67.4 | 84 | 50.6 | 21 | 51.2 | 2 | 28.6 | 8.888 | **0.031** |
|  | Reference text-books | 33 | 38.4 | 54 | 32.5 | 9 | 22.0 | 2 | 28.6 | 3.468 | 0.325 |
| In a patient applying to your clinic with severe low back pain that has affected their quality of life with no red-flag signs (radiculopathy, bladder problems, deficits etc.) detected during examination, what is your FIRST step? | Obtain X-ray/CT | 18 | 20.9 | 41 | 24.7 | 8 | 19.5 | 2 | 28.6 | 18.244 | 0.108 |
|  | Obtain MRI | 27 | 31.4 | 66 | 39.8 | 16 | 39.0 | 0 | 0.0 |  |  |
|  | Prescribe medication | 31 | 36.0 | 47 | 28.3 | 13 | 31.7 | 2 | 28.6 |  |  |
|  | Suggest bed-rest | 5 | 5.8 | 7 | 4.2 | 3 | 7.3 | 1 | 14.3 |  |  |
|  | None | 5 | 5.8 | 5 | 3.0 | 1 | 2.4 | 2 | 28.6 |  |  |
| Do you believe the choice of surgical approach (anterior, posterior, or combined anterior posterior) improve clinical outcomes in patients with thoracic and lumbar fractures? | Always | 29 | 33.7 | 71 | 42.8 | 14 | 34.1 | 2 | 28.6 | 8.735 | 0.462 |
|  | Sometimes | 50 | 58.1 | 84 | 50.6 | 24 | 58.5 | 5 | 71.4 |  |  |
|  | Never | 3 | 3.5 | 3 | 1.8 | 3 | 7.3 | 0 | 0.0 |  |  |
|  | Not Sure | 4 | 4.7 | 8 | 4.8 | 0 | 0.0 | 0 | 0.0 |  |  |
| Do you believe the addition of arthrodesis to instrumented fixation improve outcomes in patients with thoracic and lumbar burst fractures? | Always | 23 | 26.7 | 54 | 32.5 | 12 | 29.3 | 2 | 28.6 | 9.463 | 0.396 |
|  | Sometimes | 54 | 62.8 | 97 | 58.4 | 22 | 53.7 | 3 | 42.9 |  |  |
|  | Never | 2 | 2.3 | 4 | 2.4 | 4 | 9.8 | 1 | 14.3 |  |  |
|  | Not Sure | 7 | 8.1 | 11 | 6.6 | 3 | 7.3 | 1 | 14.3 |  |  |
| Do you treat low BMD (Bone Mineral Density T <-2.5) before spine surgery? | Always | 21 | 24.4 | 50 | 30.1 | 7 | 17.1 | 5 | 71.4 | 13.845 | 0.128 |
|  | Sometimes | 47 | 54.7 | 82 | 49.4 | 28 | 68.3 | 1 | 14.3 |  |  |
|  | Never | 12 | 14.0 | 21 | 12.7 | 3 | 7.3 | 0 | 0.0 |  |  |
|  | Not Sure | 6 | 7.0 | 13 | 7.8 | 3 | 7.3 | 1 | 14.3 |  |  |
| Do you prescribe high dose methylprednisolone to patients presenting with acute spinal cord injury with neurological findings? | Always | 32 | 37.2 | 56 | 34.1 | 12 | 29.3 | 0 | 0.0 | 7.712 | 0.563 |
|  | Sometimes | 29 | 33.7 | 58 | 35.4 | 15 | 36.6 | 3 | 42.9 |  |  |
|  | Never | 21 | 24.4 | 46 | 28.0 | 13 | 31.7 | 3 | 42.9 |  |  |
|  | Not Sure | 4 | 4.7 | 4 | 2.4 | 1 | 2.4 | 1 | 14.3 |  |  |
| Do you perform fusion in patients treated for lumbar stenosis with or without spondylolisthesis after decompression? | Always | 13 | 15.1 | 34 | 20.5 | 7 | 17.1 | 0 | 0.0 | 6.391 | 0.700 |
|  | Sometimes | 62 | 72.1 | 109 | 65.7 | 27 | 65.9 | 7 | 100.0 |  |  |
|  | Never | 7 | 8.1 | 18 | 10.8 | 6 | 14.6 | 0 | 0.0 |  |  |
|  | Not Sure | 4 | 4.7 | 5 | 3.0 | 1 | 2.4 | 0 | 0.0 |  |  |
| Which of the following statements about the surgical treatment of cervical radiculopathy do you agree with the most? | When correcting cervical sagittal alignment, ACDF alone is sufficient. | 26 | 30.2 | 63 | 38.0 | 11 | 26.8 | 0 | 0.0 | 16.302 | 0.061 |
|  | Anterior surgery results in better outcomes than posterior surgery | 28 | 32.6 | 47 | 28.3 | 13 | 31.7 | 1 | 14.3 |  |  |
|  | TDA results in better outcomes than ACDF when treating single level soft disc herniations. | 13 | 15.1 | 18 | 10.8 | 6 | 14.6 | 4 | 57.1 |  |  |
|  | The Neck Disability Index, SF-36, SF-12 and VAS are recommended outcome measures for assessing ... | 19 | 22.1 | 38 | 22.9 | 11 | 26.8 | 2 | 28.6 |  |  |
| What is your recommendation to a patient with unilateral extruded disc herniation with no leg pain at the time of your examination? | Microdiscectomy | 17 | 19.8 | 23 | 13.9 | 4 | 9.8 | 3 | 42.9 | 12.411 | 0.191 |
|  | Physical therapy | 29 | 33.7 | 52 | 31.3 | 12 | 29.3 | 0 | 0.0 |  |  |
|  | Medical therapy | 27 | 31.4 | 61 | 36.7 | 21 | 51.2 | 3 | 42.9 |  |  |
|  | No treatment | 13 | 15.1 | 30 | 18.1 | 4 | 9.8 | 1 | 14.3 |  |  |

**Table 16. Comparison of Years of Medical Practice in Various Clinical Scenarios**

|  | | **Years of Medical Practice (total)** | | | | | | | | | | **X^2^** | **p** |
| --- | --- | --- | --- | --- | --- | --- | --- | --- | --- | --- | --- | --- | --- |
|  |  | **Currently Training** | | **0-5** | | **6-10** | | **11-15** | | **15>** | |  |  |
|  |  | **n** | **%** | **n** | **%** | **n** | **%** | **n** | **%** | **n** | **%** |  |  |
| Do you adhere to any guidelines or scoring systems when making decisions in spinal pathologies? | Always | 16 | 50.0 | 37 | 51.4 | 27 | 48.2 | 27 | 43.5 | 25 | 34.2 | 12.209 | 0.142 |
|  | Frequently | 16 | 50.0 | 33 | 45.8 | 28 | 50.0 | 29 | 46.8 | 45 | 61.6 |  |  |
|  | Rarely | 0 | 0.0 | 2 | 2.8 | 1 | 1.8 | 6 | 9.7 | 3 | 4.1 |  |  |
| In patients where no guideline or scoring system is used, what is your decision mostly based on? | Personal experience | 14 | 42.4 | 35 | 47.3 | 29 | 51.8 | 29 | 46.8 | 41 | 54.7 | 1.904 | 0.753 |
|  | Face-to-face consultation with neurosurgeons | 18 | 54.5 | 33 | 44.6 | 28 | 50.0 | 22 | 35.5 | 32 | 42.7 | 4.177 | 0.383 |
|  | Social media collaboration with neurosurgeons | 7 | 21.2 | 5 | 6.8 | 4 | 7.1 | 18 | 29.0 | 7 | 9.3 | 20.207 | **0.000** |
|  | Literature search | 22 | 66.7 | 38 | 51.4 | 35 | 62.5 | 33 | 53.2 | 37 | 49.3 | 4.537 | 0.338 |
|  | Reference text-books | 13 | 39.4 | 27 | 36.5 | 17 | 30.4 | 24 | 38.7 | 17 | 22.7 | 5.745 | 0.219 |
| In a patient applying to your clinic with severe low back pain that has affected their quality of life with no red-flag signs (radiculopathy, bladder problems, deficits etc.) detected during examination, what is your FIRST step? | Obtain X-ray/CT | 15 | 45.5 | 20 | 27.0 | 9 | 16.1 | 12 | 19.4 | 13 | 17.3 | 28.649 | **0.026** |
|  | Obtain MRI | 7 | 21.2 | 21 | 28.4 | 22 | 39.3 | 24 | 38.7 | 35 | 46.7 |  |  |
|  | Prescribe medication | 5 | 15.2 | 26 | 35.1 | 22 | 39.3 | 22 | 35.5 | 18 | 24.0 |  |  |
|  | Suggest bed-rest | 4 | 12.1 | 3 | 4.1 | 1 | 1.8 | 2 | 3.2 | 6 | 8.0 |  |  |
|  | None | 2 | 6.1 | 4 | 5.4 | 2 | 3.6 | 2 | 3.2 | 3 | 4.0 |  |  |
| Do you believe the choice of surgical approach (anterior, posterior, or combined anterior posterior) improve clinical outcomes in patients with thoracic and lumbar fractures? | Always | 19 | 57.6 | 25 | 33.8 | 23 | 41.1 | 16 | 25.8 | 33 | 44.0 | 22.097 | **0.036** |
|  | Sometimes | 13 | 39.4 | 41 | 55.4 | 32 | 57.1 | 38 | 61.3 | 39 | 52.0 |  |  |
|  | Never | 0 | 0.0 | 2 | 2.7 | 0 | 0.0 | 4 | 6.5 | 3 | 4.0 |  |  |
|  | Not Sure | 1 | 3.0 | 6 | 8.1 | 1 | 1.8 | 4 | 6.5 | 0 | 0.0 |  |  |
| Do you believe the addition of arthrodesis to instrumented fixation improve outcomes in patients with thoracic and lumbar burst fractures? | Always | 7 | 21.2 | 25 | 33.8 | 15 | 26.8 | 20 | 32.3 | 24 | 32.0 | 18.785 | 0.094 |
|  | Sometimes | 25 | 75.8 | 38 | 51.4 | 36 | 64.3 | 34 | 54.8 | 43 | 57.3 |  |  |
|  | Never | 0 | 0.0 | 4 | 5.4 | 0 | 0.0 | 6 | 9.7 | 1 | 1.3 |  |  |
|  | Not Sure | 1 | 3.0 | 7 | 9.5 | 5 | 8.9 | 2 | 3.2 | 7 | 9.3 |  |  |
| Do you treat low BMD (Bone Mineral Density T <-2.5) before spine surgery? | Always | 13 | 39.4 | 24 | 32.4 | 9 | 16.1 | 19 | 30.6 | 18 | 24.0 | 13.544 | 0.331 |
|  | Sometimes | 12 | 36.4 | 33 | 44.6 | 35 | 62.5 | 35 | 56.5 | 43 | 57.3 |  |  |
|  | Never | 4 | 12.1 | 10 | 13.5 | 8 | 14.3 | 4 | 6.5 | 10 | 13.3 |  |  |
|  | Not Sure | 4 | 12.1 | 7 | 9.5 | 4 | 7.1 | 4 | 6.5 | 4 | 5.3 |  |  |
| Do you prescribe high dose methylprednisolone to patients presenting with acute spinal cord injury with neurological findings? | Always | 14 | 42.4 | 29 | 39.2 | 16 | 28.6 | 22 | 36.7 | 19 | 25.3 | 10.609 | 0.563 |
|  | Sometimes | 11 | 33.3 | 27 | 36.5 | 21 | 37.5 | 22 | 36.7 | 24 | 32.0 |  |  |
|  | Never | 7 | 21.2 | 15 | 20.3 | 18 | 32.1 | 14 | 23.3 | 29 | 38.7 |  |  |
|  | Not Sure | 1 | 3.0 | 3 | 4.1 | 1 | 1.8 | 2 | 3.3 | 3 | 4.0 |  |  |
| Do you perform fusion in patients treated for lumbar stenosis with or without spondylolisthesis after decompression? | Always | 7 | 21.2 | 15 | 20.3 | 6 | 10.7 | 15 | 24.2 | 11 | 14.7 | 9.053 | 0.698 |
|  | Sometimes | 23 | 69.7 | 50 | 67.6 | 39 | 69.6 | 40 | 64.5 | 53 | 70.7 |  |  |
|  | Never | 1 | 3.0 | 7 | 9.5 | 8 | 14.3 | 6 | 9.7 | 9 | 12.0 |  |  |
|  | Not Sure | 2 | 6.1 | 2 | 2.7 | 3 | 5.4 | 1 | 1.6 | 2 | 2.7 |  |  |
| Which of the following statements about the surgical treatment of cervical radiculopathy do you agree with the most? | When correcting cervical sagittal alignment, ACDF alone is sufficient. | 7 | 21.2 | 25 | 33.8 | 16 | 28.6 | 25 | 40.3 | 27 | 36.0 | 12.356 | 0.418 |
|  | Anterior surgery results in better outcomes than posterior surgery | 8 | 24.2 | 26 | 35.1 | 13 | 23.2 | 18 | 29.0 | 24 | 32.0 |  |  |
|  | TDA results in better outcomes than ACDF when treating single level soft disc herniations. | 7 | 21.2 | 7 | 9.5 | 11 | 19.6 | 8 | 12.9 | 8 | 10.7 |  |  |
|  | The Neck Disability Index, SF-36, SF-12 and VAS are recommended outcome measures for assessing ... | 11 | 33.3 | 16 | 21.6 | 16 | 28.6 | 11 | 17.7 | 16 | 21.3 |  |  |
| What is your recommendation to a patient with unilateral extruded disc herniation with no leg pain at the time of your examination? | Microdiscectomy | 6 | 18.2 | 11 | 14.9 | 9 | 16.1 | 10 | 16.1 | 11 | 14.7 | 12.596 | 0.399 |
|  | Physical therapy | 15 | 45.5 | 15 | 20.3 | 19 | 33.9 | 17 | 27.4 | 27 | 36.0 |  |  |
|  | Medical therapy | 8 | 24.2 | 33 | 44.6 | 21 | 37.5 | 27 | 43.5 | 23 | 30.7 |  |  |
|  | No treatment | 4 | 12.1 | 15 | 20.3 | 7 | 12.5 | 8 | 12.9 | 14 | 18.7 |  |  |

**Table 17. Comparison of Training in Various Clinical Scenarios**

|  | | **Have you received additional training/education/fellowship/subspecialty for Spine Surgery?** | | | | **X^2^** | **p** |
| --- | --- | --- | --- | --- | --- | --- | --- |
|  |  | **Yes** | | **No** | |  |  |
|  |  | **n** | **%** | **n** | **%** |  |  |
| Do you adhere to any guidelines or scoring systems when making decisions in spinal pathologies? | Always | 61 | 51.3 | 71 | 40.3 | 7.616 | **0.020** |
|  | Frequently | 57 | 47.9 | 94 | 53.4 |  |  |
|  | Rarely | 1 | 0.8 | 11 | 6.3 |  |  |
| In patients where no guideline or scoring system is used, what is your decision mostly based on? | Personal experience | 65 | 54.2 | 83 | 46.1 | 1.869 | 0.172 |
|  | Face-to-face consultation with neurosurgeons | 51 | 42.5 | 82 | 45.6 | 0.272 | 0.602 |
|  | Social media collaboration with neurosurgeons | 19 | 15.8 | 22 | 12.2 | 0.519 | 0.471 |
|  | Literature search | 68 | 56.7 | 97 | 53.9 | 0.224 | 0.636 |
|  | Reference text-books | 37 | 30.8 | 61 | 33.9 | 0.306 | 0.580 |
| In a patient applying to your clinic with severe low back pain that has affected their quality of life with no red-flag signs (radiculopathy, bladder problems, deficits etc.) detected during examination, what is your FIRST step? | Obtain X-ray/CT | 23 | 19.2 | 46 | 25.6 | 5.371 | 0.251 |
|  | Obtain MRI | 51 | 42.5 | 58 | 32.2 |  |  |
|  | Prescribe medication | 34 | 28.3 | 59 | 32.8 |  |  |
|  | Suggest bed-rest | 5 | 4.2 | 11 | 6.1 |  |  |
|  | None | 7 | 5.8 | 6 | 3.3 |  |  |
| Do you believe the choice of surgical approach (anterior, posterior, or combined anterior posterior) improve clinical outcomes in patients with thoracic and lumbar fractures? | Always | 50 | 41.7 | 66 | 36.7 | 1.147 | 0.803 |
|  | Sometimes | 61 | 50.8 | 102 | 56.7 |  |  |
|  | Never | 4 | 3.3 | 5 | 2.8 |  |  |
|  | Not Sure | 5 | 4.2 | 7 | 3.9 |  |  |
| Do you believe the addition of arthrodesis to instrumented fixation improve outcomes in patients with thoracic and lumbar burst fractures? | Always | 40 | 33.3 | 51 | 28.3 | 7.101 | 0.069 |
|  | Sometimes | 61 | 50.8 | 115 | 63.9 |  |  |
|  | Never | 7 | 5.8 | 4 | 2.2 |  |  |
|  | Not Sure | 12 | 10.0 | 10 | 5.6 |  |  |
| Do you treat low BMD (Bone Mineral Density T <-2.5) before spine surgery? | Always | 32 | 26.7 | 51 | 28.3 | 1.622 | 0.654 |
|  | Sometimes | 68 | 56.7 | 90 | 50.0 |  |  |
|  | Never | 12 | 10.0 | 24 | 13.3 |  |  |
|  | Not Sure | 8 | 6.7 | 15 | 8.3 |  |  |
| Do you prescribe high dose methylprednisolone to patients presenting with acute spinal cord injury with neurological findings? | Always | 35 | 29.4 | 65 | 36.3 | 3.245 | 0.352 |
|  | Sometimes | 49 | 41.2 | 56 | 31.3 |  |  |
|  | Never | 31 | 26.1 | 52 | 29.1 |  |  |
|  | Not Sure | 4 | 3.4 | 6 | 3.4 |  |  |
| Do you perform fusion in patients treated for lumbar stenosis with or without spondylolisthesis after decompression? | Always | 20 | 16.7 | 34 | 18.9 | 4.530 | 0.209 |
|  | Sometimes | 81 | 67.5 | 124 | 68.9 |  |  |
|  | Never | 17 | 14.2 | 14 | 7.8 |  |  |
|  | Not Sure | 2 | 1.7 | 8 | 4.4 |  |  |
| Which of the following statements about the surgical treatment of cervical radiculopathy do you agree with the most? | When correcting cervical sagittal alignment, ACDF alone is sufficient. | 44 | 36.7 | 56 | 31.1 | 1.037 | 0.792 |
|  | Anterior surgery results in better outcomes than posterior surgery | 34 | 28.3 | 55 | 30.6 |  |  |
|  | TDA results in better outcomes than ACDF when treating single level soft disc herniations. | 16 | 13.3 | 25 | 13.9 |  |  |
|  | The Neck Disability Index, SF-36, SF-12 and VAS are recommended outcome measures for assessing ... | 26 | 21.7 | 44 | 24.4 |  |  |
| What is your recommendation to a patient with unilateral extruded disc herniation with no leg pain at the time of your examination? | Microdiscectomy | 11 | 9.2 | 36 | 20.0 | 13.514 | **0.004** |
|  | Physical therapy | 33 | 27.5 | 60 | 33.3 |  |  |
|  | Medical therapy | 48 | 40.0 | 64 | 35.6 |  |  |
|  | No treatment | 28 | 23.3 | 20 | 11.1 |  |  |

**Table 18. Comparison of Gender in Responses to Validity and Radomization**

|  | **Gender** | | | | **t** | **p** |
| --- | --- | --- | --- | --- | --- | --- |
|  | **Male** | | **Female** | |  |  |
|  | **Mean** | **sd** | **Mean** | **sd** |  |  |
| **VALIDITY of the study designs** |  |  |  |  |  |  |
| Opinion/Commentary | 3.44 | 1.13 | 3.19 | 1.17 | 1.235 | 0.218 |
| Case Report | 3.75 | 1.00 | 3.67 | 1.01 | 0.488 | 0.626 |
| Experimental Animal Study | 3.67 | 0.97 | 3.53 | 0.97 | 0.827 | 0.409 |
| Retrospective Case Series | 4.04 | 0.81 | 4.00 | 0.79 | 0.289 | 0.773 |
| Prospective Case Series | 4.29 | 0.80 | 4.53 | 0.61 | -1.732 | 0.084 |
| Observational Study | 4.27 | 0.77 | 4.14 | 0.93 | 0.926 | 0.355 |
| Randomized Controlled Trial | 4.58 | 0.76 | 4.53 | 0.77 | 0.356 | 0.722 |
| Systematic Review and Meta-Analysis of RCT | 4.55 | 0.82 | 4.44 | 0.94 | 0.735 | 0.463 |
| **Most effective way to achieve RANDOMIZATION** |  |  |  |  |  |  |
| Randomized computer algorithm | 3.25 | 1.12 | 2.97 | 1.13 | 1.377 | 0.170 |
| Use date of birth | 3.22 | 1.06 | 3.00 | 1.12 | 1.178 | 0.240 |
| Use alternate days | 3.48 | 0.98 | 3.22 | 1.10 | 1.466 | 0.144 |
| Use patient’s ID number | 3.67 | 1.05 | 3.44 | 1.32 | 0.986 | 0.330 |
| Coin Toss | 4.53 | 0.76 | 4.39 | 0.84 | 1.063 | 0.289 |
| Use Patient’s Preference | 2.74 | 1.28 | 2.89 | 1.19 | -0.648 | 0.517 |
| Use Surgeon’s Preference | 2.84 | 1.35 | 2.92 | 1.16 | -0.343 | 0.733 |
| Randomization by the researcher | 3.67 | 1.26 | 3.44 | 1.25 | 1.014 | 0.312 |
| **Most effective technique to conceal randomization** |  |  |  |  |  |  |
| Call a separate center via telephone to obtain the next patient allocation | 3.74 | 1.00 | 3.61 | 0.96 | 0.723 | 0.470 |
| Use opaque envelopes that contain the next treatment allocation | 3.94 | 0.93 | 3.72 | 0.91 | 1.298 | 0.195 |
| Only tell the research assistant/study nurse what the next allocation will be | 3.48 | 1.07 | 3.22 | 1.12 | 1.372 | 0.171 |
| Post the randomization schedule on a board in the operating room | 3.40 | 1.13 | 3.00 | 1.29 | 1.962 | 0.051 |
| Only tell the operating surgeon what the full randomization schedule is | 3.32 | 1.25 | 3.00 | 1.26 | 1.447 | 0.149 |

**Table 19. Comparison of Age in Responses to Validity and Radomization**

|  | **Age (years)** | | | | | | | | **F** | **p** |
| --- | --- | --- | --- | --- | --- | --- | --- | --- | --- | --- |
|  | **≤30** | | **31-40** | | **41-50** | | **50>** | |  |  |
|  | **Mean** | **sd** | **Mean** | **sd** | **Mean** | **sd** | **Mean** | **sd** |  |  |
| **VALIDITY of the study designs** |  |  |  |  |  |  |  |  |  |  |
| Opinion/Commentary | 3.44 | 1.29 | 3.46 | 1.00 | 3.26 | 1.22 | 3.56 | 1.23 | 0.827 | 0.480 |
| Case Report | 3.79 | 1.10 | 3.78 | 0.86 | 3.70 | 1.05 | 3.68 | 1.25 | 0.192 | 0.902 |
| Experimental Animal Study | 3.74 | 0.94 | 3.66 | 0.88 | 3.65 | 1.14 | 3.56 | 0.95 | 0.237 | 0.870 |
| Retrospective Case Series | 4.21 | 0.80 | 4.04 | 0.75 | 4.09 | 0.88 | 3.78 | 0.85 | 2.045 | 0.108 |
| Prospective Case Series | 4.18 | 0.82 | 4.38 | 0.62 | 4.29 | 1.00 | 4.27 | 0.74 | 0.818 | 0.485 |
| Observational Study | 4.26 | 0.91 | 4.28 | 0.67 | 4.23 | 0.96 | 4.22 | 0.69 | 0.081 | 0.970 |
| Randomized Controlled Trial | 4.44 | 0.85 | 4.63 | 0.61 | 4.62 | 0.88 | 4.39 | 0.83 | 1.609 | 0.187 |
| Systematic Review and Meta-Analysis of RCT | 4.56 | 0.85 | 4.67 | 0.62 | 4.46 | 0.96 | 4.22 | 1.06 | 3.559 | **0.015** |
| **Most effective way to achieve RANDOMIZATION** |  |  |  |  |  |  |  |  |  |  |
| Randomized computer algorithm | 3.26 | 1.19 | 3.32 | 1.08 | 3.10 | 1.14 | 3.05 | 1.16 | 1.011 | 0.388 |
| Use date of birth | 3.28 | 1.10 | 3.22 | 1.06 | 3.20 | 1.08 | 3.05 | 1.07 | 0.360 | 0.782 |
| Use alternate days | 3.38 | 0.99 | 3.44 | 0.97 | 3.54 | 1.03 | 3.37 | 1.02 | 0.361 | 0.781 |
| Use patient’s ID number | 3.67 | 1.24 | 3.55 | 1.04 | 3.73 | 1.07 | 3.76 | 1.16 | 0.662 | 0.576 |
| Coin Toss | 4.72 | 0.60 | 4.46 | 0.76 | 4.51 | 0.86 | 4.54 | 0.74 | 1.184 | 0.316 |
| Use Patient’s Preference | 2.69 | 1.34 | 2.83 | 1.37 | 2.72 | 1.15 | 2.68 | 1.13 | 0.238 | 0.870 |
| Use Surgeon’s Preference | 3.05 | 1.43 | 2.87 | 1.38 | 2.72 | 1.20 | 2.88 | 1.33 | 0.575 | 0.632 |
| Randomization by the researcher | 3.59 | 1.25 | 3.66 | 1.23 | 3.59 | 1.35 | 3.76 | 1.18 | 0.198 | 0.897 |
| **Most effective technique to conceal randomization** |  |  |  |  |  |  |  |  |  |  |
| Call a separate center via telephone to obtain the next patient allocation | 3.56 | 0.94 | 3.76 | 0.96 | 3.68 | 1.10 | 3.83 | 0.92 | 0.600 | 0.615 |
| Use opaque envelopes that contain the next treatment allocation | 4.03 | 0.87 | 3.90 | 0.94 | 3.89 | 0.96 | 3.88 | 0.90 | 0.236 | 0.871 |
| Only tell the research assistant/study nurse what the next allocation will be | 3.44 | 1.07 | 3.38 | 1.13 | 3.44 | 0.97 | 3.76 | 1.09 | 1.319 | 0.268 |
| Post the randomization schedule on a board in the operating room | 3.21 | 1.24 | 3.32 | 1.18 | 3.43 | 1.14 | 3.46 | 1.05 | 0.485 | 0.693 |
| Only tell the operating surgeon what the full randomization schedule is | 3.36 | 1.31 | 3.20 | 1.28 | 3.21 | 1.24 | 3.63 | 1.11 | 1.410 | 0.240 |

**Table 20. Comparison of Regions in Responses to Validity and Radomization**

|  | **Region of practice** | | | | | | | | | | **F** | **p** |
| --- | --- | --- | --- | --- | --- | --- | --- | --- | --- | --- | --- | --- |
|  | **North America** | | **South America** | | **Europe** | | **Asia** | | **Africa** | |  |  |
|  | **Mean** | **sd** | **Mean** | **sd** | **Mean** | **sd** | **Mean** | **sd** | **Mean** | **sd** |  |  |
| **VALIDITY of the study designs** |  |  |  |  |  |  |  |  |  |  |  |  |
| Opinion/Commentary | 3.26 | 0.99 | 3.12 | 1.17 | 3.27 | 1.09 | 3.51 | 1.15 | 3.65 | 1.09 | 1.598 | 0.175 |
| Case Report | 3.79 | 0.63 | 3.61 | 1.14 | 3.42 | 1.03 | 3.85 | 0.99 | 3.92 | 0.98 | 2.530 | **0.041** |
| Experimental Animal Study | 3.74 | 0.81 | 3.70 | 0.98 | 3.63 | 0.88 | 3.60 | 1.01 | 3.78 | 1.03 | 0.335 | 0.855 |
| Retrospective Case Series | 4.42 | 0.51 | 3.85 | 0.76 | 4.03 | 0.71 | 4.07 | 0.86 | 3.89 | 0.91 | 1.874 | 0.115 |
| Prospective Case Series | 4.53 | 0.51 | 4.03 | 0.92 | 4.25 | 0.73 | 4.38 | 0.76 | 4.32 | 0.91 | 1.817 | 0.125 |
| Observational Study | 4.26 | 0.45 | 4.18 | 0.81 | 4.33 | 0.68 | 4.25 | 0.81 | 4.19 | 1.00 | 0.279 | 0.891 |
| Randomized Controlled Trial | 4.74 | 0.45 | 4.48 | 0.91 | 4.67 | 0.63 | 4.58 | 0.75 | 4.35 | 0.92 | 1.360 | 0.248 |
| Systematic Review and Meta-Analysis of RCT | 4.68 | 0.58 | 4.45 | 0.79 | 4.57 | 0.65 | 4.55 | 0.91 | 4.46 | 0.93 | 0.331 | 0.857 |
| **Most effective way to achieve RANDOMIZATION** |  |  |  |  |  |  |  |  |  |  |  |  |
| Randomized computer algorithm | 2.95 | 1.27 | 3.18 | 1.04 | 3.33 | 1.08 | 3.19 | 1.16 | 3.32 | 1.03 | 0.553 | 0.697 |
| Use date of birth | 3.21 | 0.92 | 3.15 | 0.97 | 3.15 | 0.99 | 3.17 | 1.10 | 3.46 | 1.19 | 0.622 | 0.647 |
| Use alternate days | 3.63 | 0.90 | 3.39 | 0.86 | 3.23 | 1.00 | 3.57 | 0.96 | 3.30 | 1.20 | 1.709 | 0.148 |
| Use patient’s ID number | 3.58 | 1.12 | 3.36 | 0.96 | 3.43 | 1.14 | 3.75 | 1.08 | 3.81 | 1.10 | 1.730 | 0.143 |
| Coin Toss | 4.58 | 0.61 | 4.55 | 0.67 | 4.63 | 0.61 | 4.49 | 0.84 | 4.35 | 0.86 | 0.846 | 0.497 |
| Use Patient’s Preference | 2.63 | 1.21 | 2.64 | 1.14 | 2.52 | 1.24 | 2.82 | 1.28 | 3.14 | 1.36 | 1.582 | 0.179 |
| Use Surgeon’s Preference | 2.58 | 1.26 | 2.64 | 1.17 | 2.65 | 1.25 | 2.92 | 1.41 | 3.30 | 1.18 | 1.930 | 0.105 |
| Randomization by the researcher | 3.74 | 1.05 | 3.58 | 1.28 | 3.58 | 1.29 | 3.61 | 1.26 | 3.84 | 1.28 | 0.324 | 0.861 |
| **Most effective technique to conceal randomization** |  |  |  |  |  |  |  |  |  |  |  |  |
| Call a separate center via telephone to obtain the next patient allocation | 3.68 | 0.67 | 3.30 | 1.07 | 3.65 | 0.97 | 3.82 | 1.00 | 3.81 | 1.00 | 2.031 | 0.090 |
| Use opaque envelopes that contain the next treatment allocation | 3.74 | 0.87 | 3.79 | 0.86 | 3.70 | 0.94 | 4.06 | 0.88 | 3.84 | 1.12 | 2.145 | 0.075 |
| Only tell the research assistant/study nurse what the next allocation will be | 3.42 | 0.96 | 3.58 | 1.03 | 3.20 | 0.99 | 3.55 | 1.12 | 3.38 | 1.14 | 1.263 | 0.285 |
| Post the randomization schedule on a board in the operating room | 3.37 | 1.26 | 3.48 | 0.91 | 3.20 | 1.07 | 3.33 | 1.20 | 3.57 | 1.28 | 0.703 | 0.591 |
| Only tell the operating surgeon what the full randomization schedule is | 3.21 | 1.23 | 3.36 | 1.22 | 3.13 | 1.16 | 3.27 | 1.28 | 3.59 | 1.30 | 0.837 | 0.502 |

**Table 21. Comparison of Status in Responses to Validity and Radomization**

|  | **Status** | | | | | | | | **F** | **p** |
| --- | --- | --- | --- | --- | --- | --- | --- | --- | --- | --- |
|  | **Resident** | | **Consultant** | | **Academic** | | **Other** | |  |  |
|  | **Mean** | **sd** | **Mean** | **sd** | **Mean** | **sd** | **Mean** | **sd** |  |  |
| **VALIDITY of the study designs** |  |  |  |  |  |  |  |  |  |  |
| Opinion/Commentary | 3.39 | 1.11 | 3.47 | 1.15 | 3.27 | 1.22 | 3.47 | 0.90 | 0.421 | 0.738 |
| Case Report | 3.74 | 0.98 | 3.81 | 0.98 | 3.54 | 1.11 | 3.84 | 0.96 | 0.978 | 0.403 |
| Experimental Animal Study | 3.74 | 0.79 | 3.60 | 1.06 | 3.67 | 0.98 | 3.63 | 0.96 | 0.367 | 0.777 |
| Retrospective Case Series | 4.07 | 0.76 | 3.96 | 0.87 | 4.08 | 0.81 | 4.37 | 0.50 | 1.612 | 0.187 |
| Prospective Case Series | 4.36 | 0.74 | 4.34 | 0.81 | 4.19 | 0.77 | 4.32 | 0.82 | 0.546 | 0.651 |
| Observational Study | 4.18 | 0.79 | 4.26 | 0.80 | 4.27 | 0.84 | 4.53 | 0.51 | 1.014 | 0.387 |
| Randomized Controlled Trial | 4.54 | 0.72 | 4.59 | 0.80 | 4.58 | 0.72 | 4.58 | 0.69 | 0.081 | 0.970 |
| Systematic Review and Meta-Analysis of RCT | 4.56 | 0.77 | 4.52 | 0.91 | 4.50 | 0.80 | 4.68 | 0.58 | 0.262 | 0.853 |
| **Most effective way to achieve RANDOMIZATION** |  |  |  |  |  |  |  |  |  |  |
| Randomized computer algorithm | 3.25 | 1.09 | 3.19 | 1.18 | 3.21 | 1.07 | 3.21 | 1.03 | 0.045 | 0.987 |
| Use date of birth | 3.26 | 1.10 | 3.17 | 1.09 | 3.15 | 0.94 | 3.21 | 1.18 | 0.157 | 0.925 |
| Use alternate days | 3.36 | 0.95 | 3.50 | 0.99 | 3.38 | 1.09 | 3.68 | 0.95 | 0.773 | 0.510 |
| Use patient’s ID number | 3.73 | 1.05 | 3.71 | 1.12 | 3.35 | 1.12 | 3.58 | 0.90 | 1.668 | 0.174 |
| Coin Toss | 4.52 | 0.78 | 4.52 | 0.78 | 4.56 | 0.75 | 4.37 | 0.68 | 0.285 | 0.836 |
| Use Patient’s Preference | 2.83 | 1.25 | 2.79 | 1.26 | 2.65 | 1.34 | 2.53 | 1.31 | 0.447 | 0.720 |
| Use Surgeon’s Preference | 3.10 | 1.25 | 2.72 | 1.34 | 2.81 | 1.37 | 2.89 | 1.41 | 1.418 | 0.237 |
| Randomization by the researcher | 3.70 | 1.18 | 3.66 | 1.25 | 3.63 | 1.31 | 3.32 | 1.49 | 0.496 | 0.685 |
| **Most effective technique to conceal randomization** |  |  |  |  |  |  |  |  |  |  |
| Call a separate center via telephone to obtain the next patient allocation | 3.65 | 0.98 | 3.89 | 0.93 | 3.63 | 1.01 | 3.00 | 1.15 | 5.213 | **0.002** |
| Use opaque envelopes that contain the next treatment allocation | 3.90 | 0.89 | 4.00 | 0.92 | 3.87 | 0.93 | 3.37 | 1.01 | 2.708 | **0.045** |
| Only tell the research assistant/study nurse what the next allocation will be | 3.44 | 1.00 | 3.52 | 1.11 | 3.33 | 1.15 | 3.37 | 1.07 | 0.448 | 0.719 |
| Post the randomization schedule on a board in the operating room | 3.32 | 1.18 | 3.37 | 1.15 | 3.31 | 1.21 | 3.53 | 0.96 | 0.193 | 0.901 |
| Only tell the operating surgeon what the full randomization schedule is | 3.33 | 1.19 | 3.27 | 1.29 | 3.33 | 1.28 | 3.05 | 1.27 | 0.284 | 0.837 |

**Table 22. Comparison of Clinical Practice Setting in Responses to Validity and Radomization**

|  | **Clinical Practice Setting** | | | | | | | | **F** | **p** |
| --- | --- | --- | --- | --- | --- | --- | --- | --- | --- | --- |
|  | **Academic Practice** | | **Hospital Employment** | | **Private Practice** | | **Other** | |  |  |
|  | **Mean** | **sd** | **Mean** | **sd** | **Mean** | **sd** | **Mean** | **sd** |  |  |
| **VALIDITY of the study designs** |  |  |  |  |  |  |  |  |  |  |
| Opinion/Commentary | 3.20 | 1.26 | 3.39 | 1.05 | 3.93 | 0.98 | 3.71 | 1.38 | 4.162 | **0.007** |
| Case Report | 3.51 | 1.12 | 3.78 | 0.95 | 4.07 | 0.88 | 3.86 | 0.90 | 3.163 | **0.025** |
| Experimental Animal Study | 3.59 | 0.93 | 3.69 | 0.97 | 3.61 | 1.12 | 3.86 | 0.69 | 0.304 | 0.822 |
| Retrospective Case Series | 3.98 | 0.89 | 4.09 | 0.75 | 3.95 | 0.89 | 4.00 | 0.58 | 0.553 | 0.646 |
| Prospective Case Series | 4.33 | 0.74 | 4.33 | 0.79 | 4.27 | 0.87 | 4.14 | 0.69 | 0.189 | 0.904 |
| Observational Study | 4.21 | 0.78 | 4.33 | 0.76 | 4.02 | 0.91 | 4.43 | 0.53 | 1.824 | 0.143 |
| Randomized Controlled Trial | 4.63 | 0.75 | 4.55 | 0.77 | 4.49 | 0.78 | 4.71 | 0.49 | 0.435 | 0.728 |
| Systematic Review and Meta-Analysis of RCT | 4.58 | 0.77 | 4.59 | 0.77 | 4.20 | 1.12 | 4.86 | 0.38 | 3.028 | **0.030** |
| **Most effective way to achieve RANDOMIZATION** |  |  |  |  |  |  |  |  |  |  |
| Randomized computer algorithm | 3.10 | 0.99 | 3.25 | 1.19 | 3.29 | 1.08 | 3.14 | 1.35 | 0.413 | 0.744 |
| Use date of birth | 3.02 | 1.12 | 3.29 | 1.02 | 3.20 | 1.10 | 3.14 | 1.35 | 1.178 | 0.318 |
| Use alternate days | 3.33 | 1.02 | 3.53 | 0.95 | 3.34 | 1.13 | 3.71 | 0.95 | 1.135 | 0.335 |
| Use patient’s ID number | 3.72 | 1.04 | 3.61 | 1.09 | 3.61 | 1.20 | 3.71 | 1.11 | 0.223 | 0.880 |
| Coin Toss | 4.49 | 0.93 | 4.51 | 0.71 | 4.59 | 0.67 | 4.57 | 0.53 | 0.160 | 0.923 |
| Use Patient’s Preference | 2.48 | 1.27 | 2.83 | 1.27 | 2.98 | 1.17 | 3.29 | 1.38 | 2.426 | 0.066 |
| Use Surgeon’s Preference | 2.64 | 1.28 | 2.90 | 1.35 | 3.00 | 1.30 | 3.57 | 1.40 | 1.664 | 0.175 |
| Randomization by the researcher | 3.45 | 1.30 | 3.68 | 1.23 | 3.85 | 1.24 | 3.86 | 1.46 | 1.159 | 0.326 |
| **Most effective technique to conceal randomization** |  |  |  |  |  |  |  |  |  |  |
| Call a separate center via telephone to obtain the next patient allocation | 3.73 | 0.96 | 3.70 | 1.00 | 3.80 | 1.03 | 3.57 | 1.13 | 0.168 | 0.918 |
| Use opaque envelopes that contain the next treatment allocation | 3.88 | 0.90 | 3.88 | 0.91 | 4.05 | 1.05 | 4.14 | 0.90 | 0.535 | 0.659 |
| Only tell the research assistant/study nurse what the next allocation will be | 3.27 | 1.05 | 3.44 | 1.10 | 3.76 | 1.02 | 4.29 | 0.49 | 3.402 | **0.018** |
| Post the randomization schedule on a board in the operating room | 3.26 | 1.16 | 3.39 | 1.13 | 3.24 | 1.26 | 4.43 | 0.53 | 2.416 | 0.067 |
| Only tell the operating surgeon what the full randomization schedule is | 3.03 | 1.24 | 3.32 | 1.25 | 3.41 | 1.22 | 4.71 | 0.49 | 4.505 | **0.004** |

**Table 23. Comparison of Years of Medical Practice in Responses to Validity and Radomization**

|  | **Years of Medical Practice (total)** | | | | | | | | | | **F** | **p** |
| --- | --- | --- | --- | --- | --- | --- | --- | --- | --- | --- | --- | --- |
|  | **Currently Training** | | **0-5** | | **6-10** | | **11-15** | | **15>** | |  |  |
|  | **Mean** | **sd** | **Mean** | **sd** | **Mean** | **sd** | **Mean** | **sd** | **Mean** | **sd** |  |  |
| **VALIDITY of the study designs** |  |  |  |  |  |  |  |  |  |  |  |  |
| Opinion/Commentary | 3.45 | 1.23 | 3.54 | 1.06 | 3.07 | 1.22 | 3.42 | 1.09 | 3.52 | 1.11 | 1.697 | 0.151 |
| Case Report | 3.88 | 0.96 | 3.76 | 0.92 | 3.52 | 0.97 | 3.84 | 0.93 | 3.76 | 1.17 | 1.005 | 0.405 |
| Experimental Animal Study | 3.79 | 0.96 | 3.69 | 0.81 | 3.50 | 0.97 | 3.65 | 1.06 | 3.68 | 1.05 | 0.545 | 0.703 |
| Retrospective Case Series | 4.30 | 0.68 | 4.01 | 0.77 | 4.02 | 0.77 | 4.03 | 0.81 | 3.96 | 0.92 | 1.082 | 0.365 |
| Prospective Case Series | 4.42 | 0.83 | 4.35 | 0.65 | 4.36 | 0.59 | 4.19 | 0.99 | 4.31 | 0.82 | 0.614 | 0.653 |
| Observational Study | 4.12 | 0.89 | 4.26 | 0.64 | 4.36 | 0.59 | 4.19 | 0.92 | 4.28 | 0.89 | 0.579 | 0.678 |
| Randomized Controlled Trial | 4.64 | 0.70 | 4.55 | 0.72 | 4.66 | 0.58 | 4.60 | 0.84 | 4.47 | 0.86 | 0.638 | 0.636 |
| Systematic Review and Meta-Analysis of RCT | 4.58 | 0.83 | 4.62 | 0.68 | 4.70 | 0.63 | 4.65 | 0.85 | 4.24 | 1.01 | 3.494 | **0.008** |
| **Most effective way to achieve RANDOMIZATION** |  |  |  |  |  |  |  |  |  |  |  |  |
| Randomized computer algorithm | 3.15 | 1.12 | 3.24 | 1.14 | 3.18 | 1.03 | 3.32 | 1.14 | 3.15 | 1.17 | 0.262 | 0.902 |
| Use date of birth | 3.18 | 1.10 | 3.32 | 1.14 | 3.07 | 0.93 | 3.19 | 1.05 | 3.17 | 1.11 | 0.464 | 0.762 |
| Use alternate days | 3.61 | 1.00 | 3.57 | 1.01 | 3.18 | 0.90 | 3.40 | 1.02 | 3.51 | 1.02 | 1.610 | 0.172 |
| Use patient’s ID number | 3.67 | 1.22 | 3.59 | 1.03 | 3.59 | 1.04 | 3.68 | 1.14 | 3.69 | 1.10 | 0.128 | 0.972 |
| Coin Toss | 4.58 | 0.83 | 4.42 | 0.81 | 4.61 | 0.59 | 4.66 | 0.63 | 4.40 | 0.90 | 1.531 | 0.193 |
| Use Patient’s Preference | 2.67 | 1.31 | 3.08 | 1.32 | 2.48 | 1.26 | 2.74 | 1.34 | 2.71 | 1.10 | 1.956 | 0.101 |
| Use Surgeon’s Preference | 3.03 | 1.40 | 3.11 | 1.29 | 2.54 | 1.35 | 2.77 | 1.36 | 2.83 | 1.27 | 1.706 | 0.149 |
| Randomization by the researcher | 3.42 | 1.44 | 3.80 | 1.06 | 3.63 | 1.36 | 3.69 | 1.29 | 3.56 | 1.25 | 0.637 | 0.636 |
| **Most effective technique to conceal randomization** |  |  |  |  |  |  |  |  |  |  |  |  |
| Call a separate center via telephone to obtain the next patient allocation | 3.58 | 1.09 | 3.96 | 0.78 | 3.64 | 0.92 | 3.69 | 1.14 | 3.64 | 1.04 | 1.480 | 0.208 |
| Use opaque envelopes that contain the next treatment allocation | 4.06 | 0.90 | 3.91 | 0.92 | 3.80 | 0.90 | 4.00 | 0.96 | 3.85 | 0.94 | 0.617 | 0.651 |
| Only tell the research assistant/study nurse what the next allocation will be | 3.18 | 1.26 | 3.54 | 1.09 | 3.25 | 1.12 | 3.52 | 1.04 | 3.59 | 0.97 | 1.488 | 0.206 |
| Post the randomization schedule on a board in the operating room | 3.18 | 1.38 | 3.43 | 1.14 | 3.25 | 1.21 | 3.35 | 1.20 | 3.43 | 1.00 | 0.451 | 0.772 |
| Only tell the operating surgeon what the full randomization schedule is | 3.15 | 1.33 | 3.39 | 1.19 | 3.00 | 1.31 | 3.19 | 1.44 | 3.52 | 1.04 | 1.705 | 0.149 |

**Table 24. Comparison of Training in Responses to Validity and Radomization**

|  | **Have you received additional training/education/fellowship/subspecialty for Spine Surgery?** | | | | **t** | **p** |
| --- | --- | --- | --- | --- | --- | --- |
|  | **Yes** | | **No** | |  |  |
|  | **Mean** | **sd** | **Mean** | **sd** |  |  |
| **VALIDITY of the study designs** |  |  |  |  |  |  |
| Opinion/Commentary | 3.37 | 1.22 | 3.44 | 1.08 | -0.581 | 0.562 |
| Case Report | 3.62 | 1.09 | 3.83 | 0.94 | -1.740 | 0.083 |
| Experimental Animal Study | 3.60 | 0.96 | 3.69 | 0.98 | -0.776 | 0.438 |
| Retrospective Case Series | 4.03 | 0.86 | 4.04 | 0.78 | -0.203 | 0.839 |
| Prospective Case Series | 4.32 | 0.77 | 4.32 | 0.79 | 0.001 | 0.999 |
| Observational Study | 4.24 | 0.82 | 4.26 | 0.77 | -0.208 | 0.835 |
| Randomized Controlled Trial | 4.53 | 0.78 | 4.59 | 0.75 | -0.684 | 0.494 |
| Systematic Review and Meta-Analysis of RCT | 4.53 | 0.85 | 4.55 | 0.82 | -0.255 | 0.799 |
| **Most effective way to achieve RANDOMIZATION** |  |  |  |  |  |  |
| Randomized computer algorithm | 3.24 | 1.17 | 3.19 | 1.09 | 0.357 | 0.722 |
| Use date of birth | 3.19 | 1.09 | 3.20 | 1.05 | -0.066 | 0.947 |
| Use alternate days | 3.41 | 1.02 | 3.48 | 0.98 | -0.591 | 0.555 |
| Use patient’s ID number | 3.70 | 1.09 | 3.61 | 1.09 | 0.735 | 0.463 |
| Coin Toss | 4.52 | 0.78 | 4.52 | 0.77 | 0.001 | 0.999 |
| Use Patient’s Preference | 2.68 | 1.28 | 2.82 | 1.27 | -0.946 | 0.345 |
| Use Surgeon’s Preference | 2.81 | 1.36 | 2.88 | 1.31 | -0.478 | 0.633 |
| Randomization by the researcher | 3.78 | 1.26 | 3.56 | 1.25 | 1.487 | 0.138 |
| **Most effective technique to conceal randomization** |  |  |  |  |  |  |
| Call a separate center via telephone to obtain the next patient allocation | 3.70 | 0.99 | 3.74 | 0.99 | -0.332 | 0.740 |
| Use opaque envelopes that contain the next treatment allocation | 4.00 | 0.85 | 3.85 | 0.97 | 1.413 | 0.159 |
| Only tell the research assistant/study nurse what the next allocation will be | 3.40 | 1.17 | 3.49 | 1.02 | -0.698 | 0.486 |
| Post the randomization schedule on a board in the operating room | 3.37 | 1.15 | 3.34 | 1.16 | 0.163 | 0.871 |
| Only tell the operating surgeon what the full randomization schedule is | 3.26 | 1.31 | 3.30 | 1.22 | -0.281 | 0.779 |
